# Microfluidic SERS devices: brightening the future of bioanalysis

**DOI:** 10.1007/s43939-022-00033-3

**Published:** 2022-12-15

**Authors:** Maria João Oliveira, Ana Dalot, Elvira Fortunato, Rodrigo Martins, Hugh J. Byrne, Ricardo Franco, Hugo Águas

**Affiliations:** 1 CENIMAT|i3N, Department of Materials Science, School of Science and Technology, NOVA University Lisbon and, CEMOP/UNINOVA, Caparica, Portugal; 2 Associate Laboratory i4HB—Institute for Health and Bioeconomy, Faculdade de Ciências e Tecnologia, Universidade NOVA de Lisboa, 2829-516 Caparica, Portugal; 3 UCIBIO—Applied Molecular Biosciences Unit, Departamento de Química, Faculdade de Ciências e Tecnologia, Universidade NOVA de Lisboa, 2829-516 Caparica, Portugal; 4 FOCAS Research Institute, Technological University Dublin, Camden Row, Dublin 8, Dublin, Ireland

## Abstract

A new avenue has opened up for applications of surface-enhanced Raman spectroscopy (SERS) in the biomedical field, mainly due to the striking advantages offered by SERS tags. SERS tags provide indirect identification of analytes with rich and highly specific spectral fingerprint information, high sensitivity, and outstanding multiplexing potential, making them very useful in in vitro and in vivo assays. The recent and innovative advances in nanomaterial science, novel Raman reporters, and emerging bioconjugation protocols have helped develop ultra-bright SERS tags as powerful tools for multiplex SERS-based detection and diagnosis applications. Nevertheless, to translate SERS platforms to real-world problems, some challenges, especially for clinical applications, must be addressed. This review presents the current understanding of the factors influencing the quality of SERS tags and the strategies commonly employed to improve not only spectral quality but the specificity and reproducibility of the interaction of the analyte with the target ligand. It further explores some of the most common approaches which have emerged for coupling SERS with microfluidic technologies, for biomedical applications. The importance of understanding microfluidic production and characterisation to yield excellent device quality while ensuring high throughput production are emphasised and explored, after which, the challenges and approaches developed to fulfil the potential that SERS-based microfluidics have to offer are described.

## Introduction

Accurate and timely detection of infections, food contaminants and pollutants, is crucial for effective response management and vigilance, as highlighted, for example, by the Severe Acute Respiratory Syndrome Human Coronavirus (SARS-CoV-2) pandemic. To date, detection modalities have relied largely on techniques used in highly specialised research laboratories, such as the Enzyme-Linked Immunosorbent Assay (ELISA) and Polymerase Chain Reaction (PCR). However, the massive advances of nanotechnology have allowed for the development of new, rapid, portable and efficient systems with applications aimed at molecular detection and imaging, multifunctional therapeutics, and prevention and control of diseases [1–4].

Early diagnosis is crucial in infectious disease management and enhances the clinical outcome. Current conventional diagnostic techniques such as the ELISA and PCR, provide reliable and sensitive detection and are capable of treatment monitoring [5]. These highly accurate diagnostic tests are available for most infectious diseases of public health importance in the developed world, but these methods are technically challenging and expensive to implement in resource-limited settings. Light microscopy-based cytological examination is easily implemented and has been the gold-standard technique for some infectious diseases (*e.g.* malaria and tuberculosis). However, it requires equipment, a source of electricity, and specialised personnel, making this technique unfeasible in rural communities that lack these resources, hindering remote testing [6].

This has driven a strong interest in the development of diagnostic platforms applicable for widespread implementation at Point-Of-Care Testing (POCT) sites. The ideal diagnostic platform adheres to several criteria defined by World Health Organization (WHO) as ASSURED (affordable, sensitive, specific, user-friendly, rapid and robust, equipment-free, and deliverable to end-users) [6]. Thus, the development of detection methods has been focused on technologies applicable in all types of healthcare systems to guide treatment and clinical management decisions.

Among the range of novel detection techniques, surface-enhanced Raman spectroscopy (SERS) has emerged as an ultrasensitive vibrational spectroscopic technique [7, 8]. Once seen as merely an extension of Raman spectroscopy, SERS has evolved as a new and vibrant multidisciplinary research field [7]. Although a field with a short history, since its serendipitous discovery [9], SERS has seen unbelievable advancements in many different areas and in several configurations. The use of SERS in bioanalysis is rapidly evolving as a suitable and powerful approach for disease detection and treatment [8].

The classical approach to SERS analysis is to fabricate a nanostructure and observe an enhanced light scattering response of an analyte molecule in the vicinity of the nanostructure, which can then be used to characterise it. However, this label-free approach is dependent on the Raman scattering efficiency of the analyte, as well as its coupling to the nanostructure. Non-specific interactions, competitive binding and/or fouling by other species can all contribute to a lack of quantitative reproducibility of the process. To increase the reliability and versatility of SERS-based biological assays, nanostructures can be functionalised with target ligands that will specifically bind to an analyte and induce an enhanced response. This response can then be coupled with a label and provide an indirect way of detection.

The development of this indirect method, employing Raman labelled immunogold system, marks the beginning of the field of SERS tags applications. In addition, the combination of SERS with other techniques such as microfluidics enables the incorporation of the high sensitivity and multiplex capability to a high throughput analysis system [7]. To this end, SERS-microfluidics systems have been extensively investigated in biomedical applications [10]. These systems are an attractive choice due to the potential impact of early detection of diseases as infections. Microfluidics consolidate sample preparation, manipulation and separation, and *in-situ* detection into one working unit without impairing reliable and consistent performance [10].

**Fig. 1:**
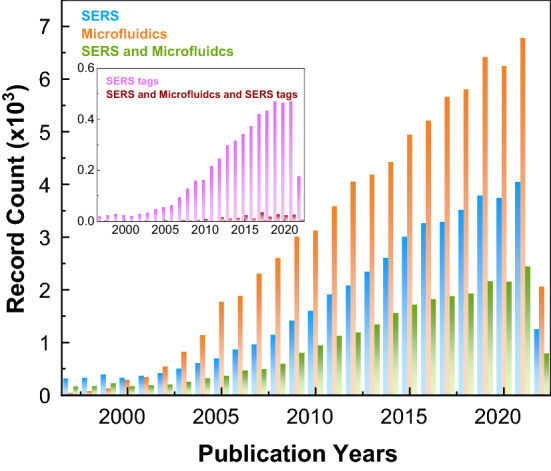
Number of publications search in Web of Science, as SERS: “TOPIC: (surface enhanced Raman) OR TOPIC: (sers)”; microfluidics: “TOPIC: (microfluidc*)”; SERS tags: “TOPIC: (surface enhanced Raman) OR TOPIC: (sers) AND TOPIC: SERS tag, OR TOPIC: SERS label OR TOPIC:SERS probe”. This search is as of June 2022 [11]

Since then, hundreds of research papers about the design and possible applications of SERS tags and microfluidics have been published, as shown by the steady growth in Fig. 1. Nevertheless, the number of publications in microfluidic SERS-related research indicates that much development is still required.

Many interesting review articles have been published concerning the applications of SERS tags [12–17] and others concerning microfluidics SERS-based device applications [18–26]. The present review is not conceived as a thorough review of applications reported in literature, but rather a summary of the key strategies to be considered when designing a SERS tag, highlighting the importance of understanding the mechanisms of the nanoparticle (NP)-biomolecule system for successful and reproducible design of bioconjugates. Additionally, it focuses on how the choice of the materials and fabrication method can influence the final outcome for a microfluidic SERS-based sensing system. Throughout the review, the advantages of combining microfluidics and SERS for biosensing are presented and analysed. Finally, the challenges and perspectives of working with SERS tags and microfluidic devices and applying them in biosensing are discussed.

## Surface-enhanced Raman spectroscopy

### Fundamental theory of surface-enhanced Raman scattering

**Fig. 2:**
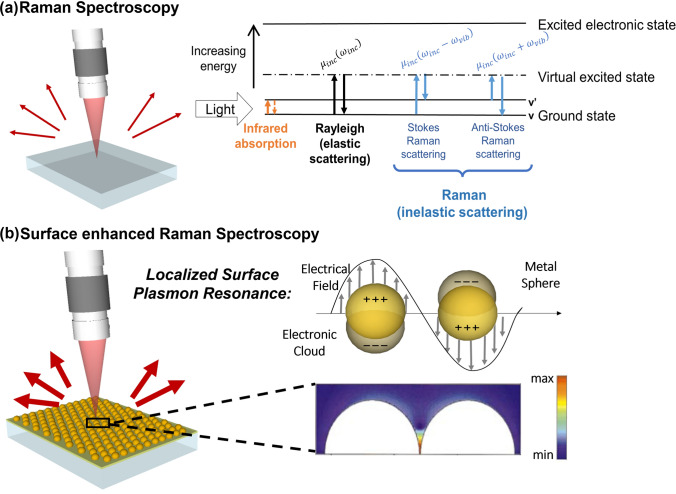
Raman vs SERS phenomenon. **a** Schematic of Raman Spectroscopy and energy diagram representing (from left to right) the infrared absorption, elastic Rayleigh scattering and the inelastic anti-Stokes (left) and Stokes (right) Raman scattering with ω_inc_, ω_inc_ ± ω_vib_ and ω_vib_ referring to the frequencies of the incident light, the Raman scattered light, and the molecular vibration, respectively. ${\upmu }_{inc}$ refers to the induced dipole moment, which in turn, is the product of Raman polarisability, α, and the magnitude of the incident electromagnetic field, $E$. **b** Illustration of SERS and of the LSPR effect. This consists of the collective oscillation of the conduction electrons in a metal NP in resonance with the frequency of the incident light. The colour plot at the bottom corresponds to the electric field intensity profile in the inter-space of a dimer with two Au nanospheres having a separation of 1 nm. The colour scale is logarithmic. Adapted from [44].

SERS is a nanoscale phenomenon that enhances the Raman signal of molecules adsorbed on metal nanostructured substrates (Fig. 2) [7, 9]. The origin of this observed enhancement has been debated since its original observation in 1974, and it is now accepted among researchers that the overall enhancement (quantified in terms of the enhancement factor—EF [27]) can arise from either and/or a combination of two mechanisms: so-called chemical (CHEM) [28] enhancement, due to charge transfer mechanisms to or from the metal particles (commonly known as metal -molecule bond), and electromagnetic (EM) [29] enhancement, associated with surface plasmon excitation in metal nanostructures.

The chemical enhancement mechanism of SERS is based on the interaction of chemisorbed molecules with the metal surface and is independent of the EM environment [30, 31]. CHEM can be attributed to two contributions: the changes in the polarisability derivative due to the transfer of charge induced by the molecule adsorbed on the metal and the lowest unoccupied molecular orbital (LUMO) to highest occupied molecular orbital (HOMO) transition of the chemisorbed molecule. This occurs when the energy of the transition falls symmetrically with the Fermi level of the metal surface and then the excitation of half the energy can make the transition. Generally, its contribution to the EF is of the order of one to three orders of magnitude [32], and significantly smaller than the EM contribution, which is commonly considered to be the dominant mechanism for SERS.

Raman scattering efficiency is related to the interaction of the EM field of the incident light, of specific wavelengths (or frequency ω), with the polarisability of the scattering bond (Fig. 2b). EM enhancement is the result of the concentration of electromagnetic energy $E(\omega )$ in the vicinity of a good, nanostructured, conductor when the incident light frequency is resonant with that of the collective oscillation of the nanostructure surface electrons, a phenomenon known as localised surface plasmon resonance (LSPR). As a result, this localised electric-field enhancement can act as an antenna, leading to highly amplified Raman scattering signals of molecules adsorbed onto, or in the vicinity of the surface of the nanostructures [7, 8, 33]. The SERS enhancement can be approximately described by $\frac{{\left|E\left({\omega }_{inc}\right)\right|}^{4}}{{\left|{E}_{0}\right|}^{4}}$, ${E}_{0}$ being the oscillating electric field of the incoming laser radiation and ${\left|E\left({\omega }_{inc}\right)\right|}^{4}\approx {{\left|E\left({\omega }_{inc}\right)\right|}^{2}\left|E\left({\omega }_{vib}\right)\right|}^{2}$, because the Stokes Raman scattered frequency (${\omega }_{vib}$) is much smaller than the incident ${\omega }_{inc}\gg {\omega }_{vib}$ or ${\omega }_{inc}\approx {{\omega }_{inc}-\omega }_{vib}$ [8].

Electric field enhancements have been observed for nanostructured surfaces and colloidal NPs [34, 35]. Enhancements depend on the size of the nanofeature [28] and are observed to be especially strong in the neighbourhood of sharp peaks, at which so called “hotspots” are produced [34, 36–38]. Hotspots are also observed when the near-field regions between adjacent nanofeatures or particles overlap [39]. In NP systems, SERS performance is optimised by controlling their shape to produce sharp structures, or by NP aggregation. Other geometrical structures such as within NP junctions and flat metal surfaces that support SPR can generate hotspots and many reports try to maximise this feature with gap distances (ideally in the range of 2–10 nm) (see Fig. 2b) [40, 41].

A persistent misconception regarding SERS EM is that the SERS excitation profile does not necessarily follow the extinction spectrum of the SERS substrate [42]. This is because the extinction spectrum of the SERS substrate includes absorptions and scattering contributions from the entire nanostructure and the SERS spectrum mainly depends on the resonances of the “hottest spots” [43]. In fact, works by Schatz and Van Duyne showed that the highest EM could be achieved away from the wavelength of maximum absorption, due to “dark” plasmon, or quadrupolar modes. These dark plasmon modes can lead to strong far-field intensities because the dipole field of the absorbed molecules can excite quadrupolar and higher-order multipolar resonances with better efficiency than light waves [7].

Ultimately, the EM depends on the nanostructure’s inherent properties (*e.g.* material, size and shape) and the CHEM is determined by the chemical features of the analytes attached to the metal surface. It should be noted that, in both cases, the SERS effect is extremely localised to the surface of the nanostructure. CHEM enhancement only applies to molecular species adsorbed to the surface, while EM enhancement decays within ~ 10 nm of the surface [7]. The surface can rapidly become saturated, limiting the sensitivity of the technique, and, in the case of complex media, competitive binding can influence the selectivity. An in-depth review of the SERS mechanisms is beyond the scope of this review and can be found elsewhere [7, 28, 30–32].

### SERS in bioanalysis: direct versus indirect detection

The confirmation of a particular condition is commonly accomplished by detection of molecular biomarkers [45]. Biomarkers, individual or combined, act as biochemical indicators of a specific state and can be used for detecting the presence or even the stage of progression of a disease [45]. Traditional diagnostic tools, such as ELISA [46], the gold standard for detection of proteins in physiological samples, PCR [47], electrophoresis [48], and fluorescence methods [49], among others, do not provide the limit of detection (LOD) required to detect the new, low abundance molecular biomarkers identified in recent fundamental biological studies [50]. This increased demand for molecular biomarker detection has instigated the development of ultrasensitive sensors based on nanotechnology.

The combination of biological, material, and instrumentation studies allowed the development of devices able to use specific biochemical reactions mediated by biorecognition ligands to detect and translate into a signal, known as biosensors. Various nanomaterials can be used as signal transducers in biosensing systems, as they provide strong signal intensities, tuneable physicochemical properties (*e.g.* LSPR and surface chemistry) and exhibit extremely large ensemble surface areas using a very small quantity of nanomaterials [7, 8, 33]. In theory, any biomarker, identified by fundamental studies, can be correlated with suitable biorecognition ligands, which, in turn, can be coupled to any transducer. The numerous possible combinations of nanomaterials and biomarkers being integrated into miniaturised devices, present biosensor platforms a versatility that benefits tremendously diagnostic tools and can provide effectively early diagnosis of life-threatening conditions [2, 51].

The interest in SERS as a bio-detection tool is due to its outstanding analytical features, such as high sensitivity, specificity, and, multiplexing and non-destructive detection abilities [7, 8, 33, 52]. Furthermore, SERS has evolved considerably and found application as a diagnostic tool for medical samples allowing the detection of biorelevant targets in complex biochemical matrices which is enabled due to the low interference from water resulting in minimal background signals from aqueous biological samples [5]. SERS substrates as part of a biosensor for bioanalysis applications can be fabricated via top-down approaches, involving patterned complex nanostructures on a surface, and bottom-up methods, utilising chemically synthesised NPs in suspension or assembled into well-defined arrays on a substrate [53]. Top-down strategies include nanolithography, that allows production of substrates such as film over nanospheres, normally used to amplify the biomarker signal onto the surface [54]. These provide high enhancements and precision in patterning ensuring a sensitive and uniform distribution. Nevertheless, these techniques are time-consuming and provide low throughput, whereas bottom-up approaches have advantages in terms of throughput, simplicity of preparation, and time effectiveness [54]. The self-organisation offered by these methods can be fabricated on non-planar substrates, or even on liquid/liquid interfaces [54]. Although associated with lack of control, is it now accepted that both state-of-art approaches present good SERS signal reproducibility, making them ideal for mass fabrication of SERS substrates [7].

**Fig. 3:**
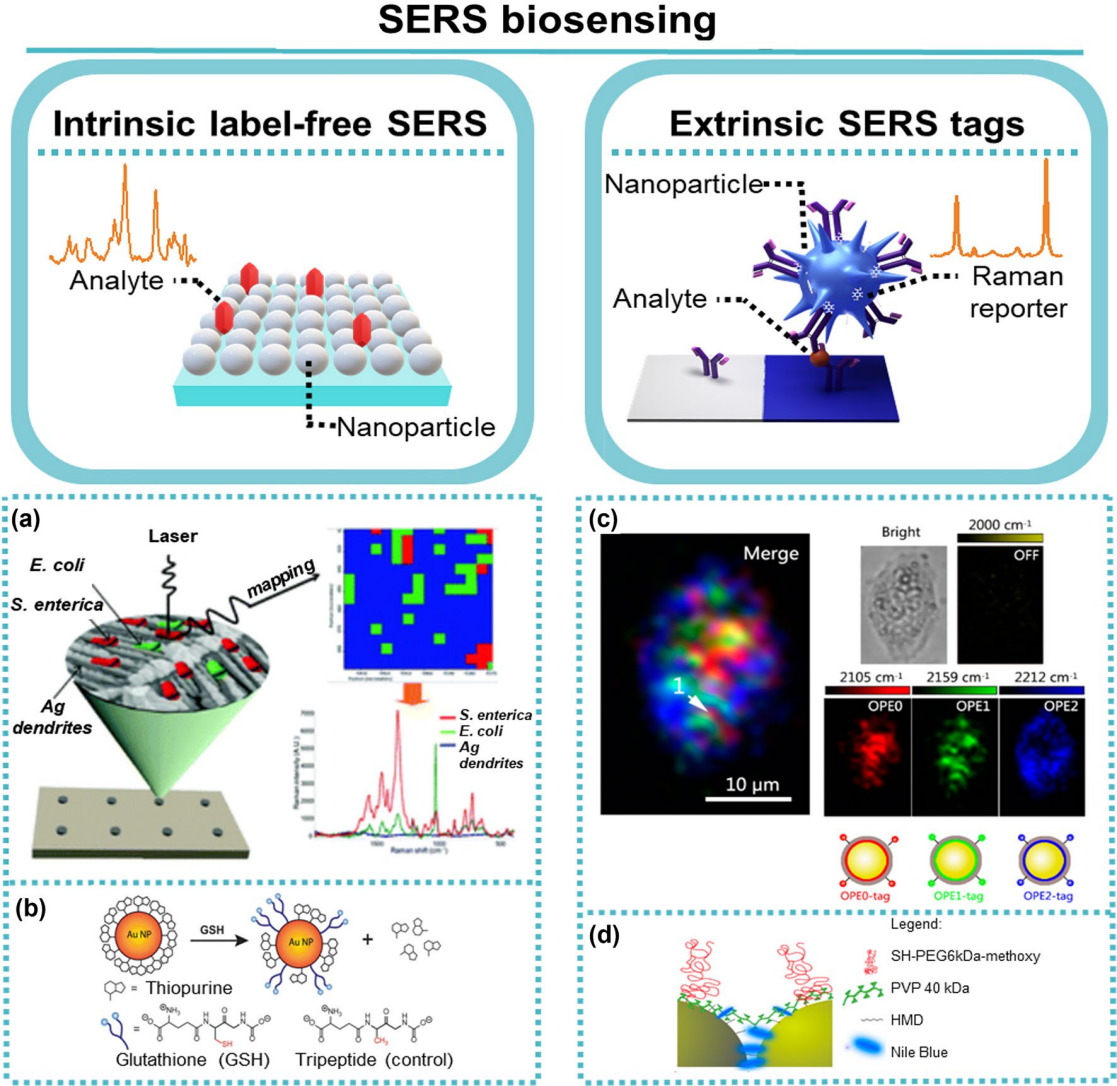
SERS biosensing. Left: direct or label-free intrinsic SERS. Right: indirect or extrinsic SERS tags. **a** Schematic details on the SERS-active substrate with Ag dendrites for simultaneous detection and identification of a bacterial mixture containing *Salmonella* and *Escherichia coli* (E. coli). Adapted from [55]. **b** Release of thiopurine on AuNPs via glutathione. Adapted from [56]. **c** Three-colour SERS imaging using SERS probes of alkyne SERS palette. Reprinted (adapted) with permission from [57]. Copyright 2016 American Chemical Society. **d** Schematic of SERS-based biotags assembly process and subsequent etching of the metallic cores by hexacyanoferrate and thiosulfate. Reprinted (adapted) with permission from [58]. Copyright 2015 American Chemical Society

Regarding the biosensing method, SERS phenomenon can be explored in two ways (Fig. 3): (i) direct or label-free intrinsic SERS and (ii) indirect or extrinsic SERS tags. The former takes profit from an enhanced Raman signal from the analyte when it is in close vicinity of a nanostructured metal surface, whereas the latter uses a priori knowledge of a Raman signal from a reporter (RR) molecule and utilises it as an amplified label for recognition of target analyte which is mediated by a target-specific ligand [5, 7, 12, 17]. Direct SERS sensing, regarded as the conventional detection mode, was the first method to be used in the SERS field, and it is still prevalent in literature, due to its simplicity and sensitivity. However, if the target is within a complex medium, overlapping of vibrational modes of different molecules is inevitable which hinders interpretation. Thus, a sensor detection method based on indirect or extrinsic SERS tags offers a more universal and versatile option towards biosensing.

### Properties of SERS-tags

In optical methods, the signal generation that allows the localisation and detection of target molecules is generally provided by labelling agents [17]. Several examples are well represented in literature, such as fluorescent dyes/Quantum Dots (QDs) (*e.g.*, in immunofluorescence [3, 59]), enzymes that catalyse a colorimetric reaction (*e.g.*, horseradish peroxidase in ELISA and immunohistochemistry [60, 61]) and Raman reporters (RRs) [7]. However, the detection event is entirely controlled by the noncovalent interactions of the target-specific ligands used for molecular recognition of the target. For instance, antibodies specifically recognise antigens, whereas single oligonucleotide strands hybridise with a matching sequence [13, 15, 16]. The *target* can be a metabolite or protein biomarker in blood or tissue or even a membrane protein on a specific cell [51].

The terms *SERS tag* and *SERS probe* will be used here to describe a core NP, or NP cluster coated with a RR and with a target specific-ligand [7, 13, 15], whereas the term *label* will be used as a synonym to reporter meaning the specific “code” that provides the identification of the recognition event. These multi-component ensembles provide a uniquely strong spectral signature with wide ranging applications in biological detection and imaging [13]. 

**Fig. 4:**
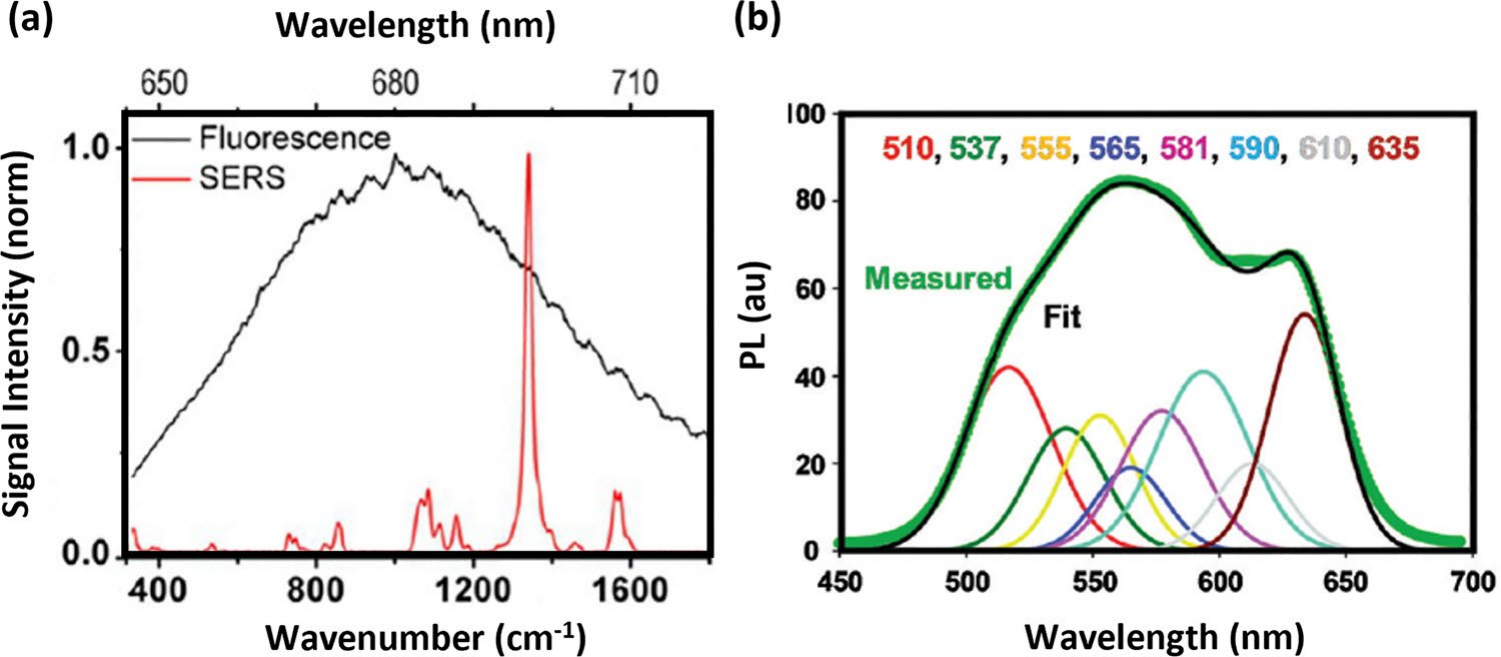
Comparison of spectral emission profiles: **a** fluorescence from Cy5 and SERS from 4-nitrothiobenzoic acid (4-NTB) on AuNPs excited with 632.8 nm laser radiation. Adapted from [69]. **b** photoluminescence spectra of QDs composite (green) with deconvolved contributions of eight different QD colours (510, 537, 555, 565, 581, 590, 610, and 635 nm QDs) and the overall model fit (black). Reprinted (adapted) with permission from [70]. Copyright 2009 American Chemical Society

Table 1 summarises and compares the features of such SERS-tags, with similar labelling techniques using quantum dots and organic dyes, while Fig. 4 shows a spectral comparison of the three types of labels.

**Table 1:** Comparison of the main features of SERS tags, conventional organic dyes, and QDs [65– 68]

	SERS tags	Dyes	QDs
Physical phenomenon	Raman scattering	Electronic absorption and fluorescence emission	Photoluminescence/fluorescence emission
Core composition	Noble metal NPs	Organic compounds	Semiconductor materials (*e.g.* ZnS, ZnSe, CdS, CdSe and others)
Size	≈60 nm	≈1 nm	≈10 nm
Spectral full-width-at-half-maximum (FMHM)	2 nm	> 50 nm	25–35 nm
Fingerprinting ability	Yes	No	No
Multiplexing capacity	> 100 (theoretically)	≈20	12–15
Photostability	Strong	Weak^1^	Mid^2^
Toxicity	No	Mild	Strong
Technology maturation/translation to medicine	Early stage	Developed	In the process of translation

^1^Degrade under weak excitation

^2^Decay under strong excitation

The advantages of SERS tags include: (i) spectral capacity for multiple detection (multiplexing) due to the narrow full width at half-maximum (FMHM) of vibrational Raman bands (≈10 cm^−1^) compared to the broad emission profiles of molecular fluorophores (≈1500 cm^−1^) (as seen in Fig. 4); (ii) quantitative ability since the SERS signal is proportional to the recognition events between analyte-ligand from the SERS tags; (iii) photostability allowing repetition or long-time measurements of the SERS tag contrary to common fluorophores; (iv) possibility of addressing spectrally distinct SERS tags with a single wavelength source; (v) use of SERS tags with plasmon resonances in the red to near-infrared (NIR) minimises autofluorescence contribution from biological tissues. Nevertheless, in studies of biological cells which require high-spatial resolution confocal microscopy, the size of SERS tags might be an impairment. Other, often overlooked challenges are related to the bioconjugation process and the dearth of standardised protocols for quality control over the colloids in the same and different batches, as well as synthesis scalability [7, 59, 62–64].

## The building blocks of SERS-tags

**Fig. 5:**
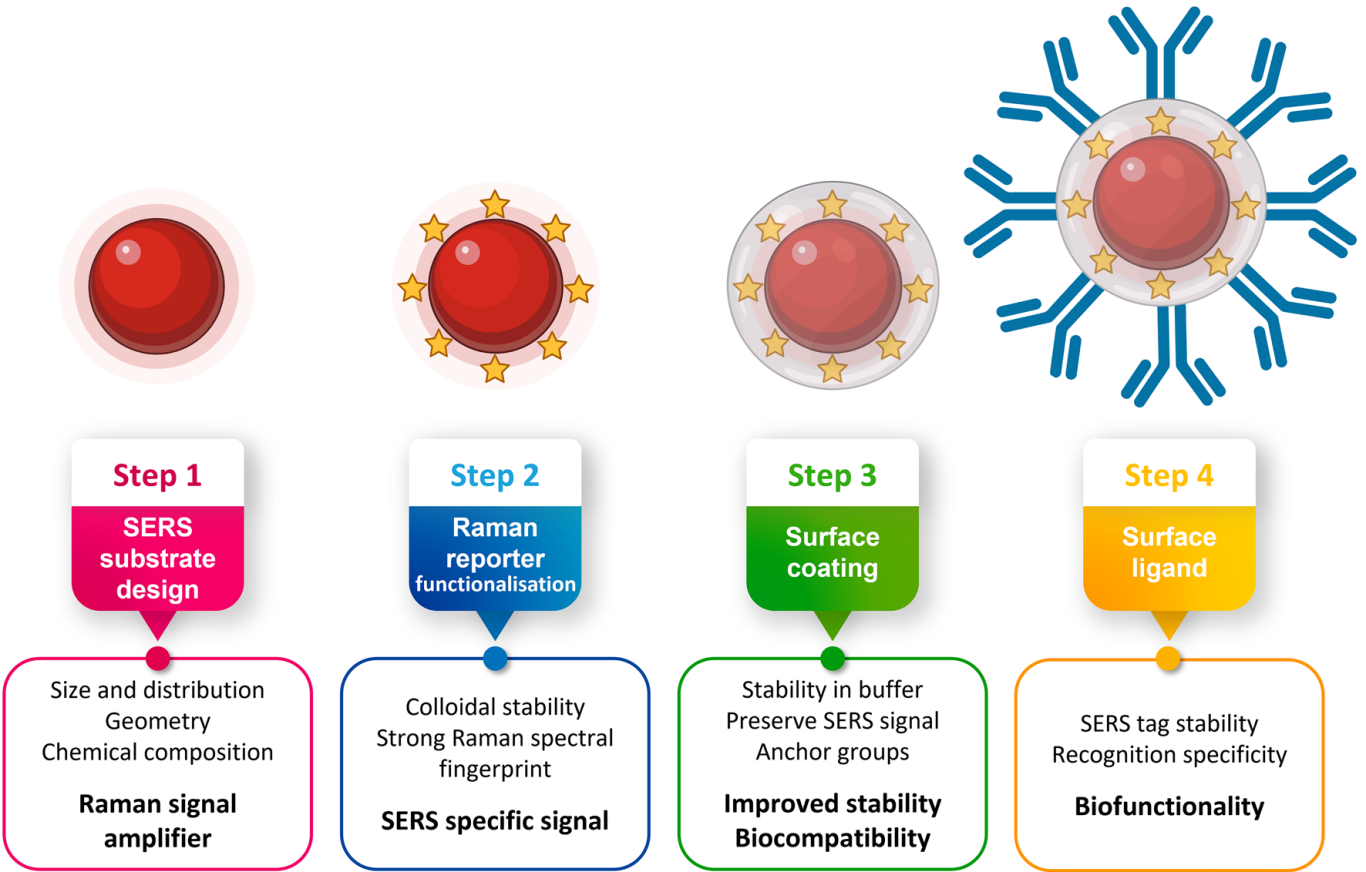
Sequential typical procedure for fabricating a SERS tag

As depicted in Fig. 5, a SERS tag comprises a noble metal NP core with RRs functionalised to the metal surface providing a spectral signature and a target-specific ligand, such as an antibody or an oligonucleotide, to enable bioanalytical and biomedical applications. Between the RR and the recognition biomolecule, a biocompatible shell can be inserted to keep the RR near the NP, to maximise SERS signal enhancement and stabilise the NPs. Each one of the components can be chosen from a large number of options, depending on the desired application: (i) the type of NP, (normally Au and Ag but other examples report the use of Cu/Mn [71], ZnO [72], W_18_O_49_ nanocrystals [73], MoO_2_ [74]_,_ graphene/MoS_2_ nanohybrids [75] etc.) as well as its size and shape (nanorods and nanostars being preferred to spheres [34]); (ii) number of NPs: single *versus* clusters (assembly of NPs offers gaps which are a source of hotspots for increased signal); (iii) the type of RR (*e.g.*, Chromophores or small aromatic thiols); (iv) the type of protective shell (*e.g.*, polymers as polyethylene glycol (PEG), biomolecules, and silica); (v) the type of bioconjugation process, depending on the functional groups available from the previous step (*e.g.* carboxylic acids, amines, thiols, etc.) [7, 12, 13, 15, 16, 42, 76, 77]. Figure 5 represents the sequential procedure for designing a SERS tag and Table 2 summarises the most common molecules that form a SERS tag.

**Table 2:** Components of SERS tags with some examples [7, 12, 13, 15, 17, 42, 63, 76–82]

SERS nanotag components Function	Type	Examples	Linking mode	Advantages	Disadvantages	Features to consider
Plasmonic nanoparticle *Plasmonic activity* Metal Size Shape	Au, Ag 20–200 Spheres, rods, stars	–	–	• Signal generation due to LSPR • Facile synthesis by bottom-up strategies	• Lack of standardised protocols • Lack of control over NP dispersity	• LSPR band location • Number of hotspots *per* particle • Synthesis: easy, reproducibility and scalable
Number of NPs		Monomer *vs* cluster				
RR molecule *Provide characteristic spectral signature for sensitive and indirect identification of the selected target*	Dyes:nitrogen-containing cationic dye Sulphur-containing dye	Crystal Violet Rhodamine B/6G Nile blue Cyanine, CV, Malachite green	Electrostatic interaction N-Au(Ag) interaction S–Au(Ag) interaction	• Plentiful fingerprint information • Commercially-available • Larger Raman scattering cross section for dyes	• Emission in fingerprint region (400–1800 cm^−1^) • Spectral overlapping susceptibility in multiplexing assays • Fluorescence for dyes • Smaller Raman scattering cross section for thiol-small aromatic molecules	• Raman scattering cross section • Surface-seeking groups • Metal affinity • Ability to form SAM • Water solubility • Reactivity • Simplicity and low-cost
	Thiol-small aromatic molecules	Thiophenol,aminohexanethiol, naphthalene dithiol, mercaptobenzoicacid, 1,4-biphenyldithiol	S–Au(Ag) interaction			
	Triple bond-containing RRs	4-Ethynylbenzenethiol derivatives 4- Mercaptobenzonitrile Metal carbonyl compounds	$C\equiv C$ $C\equiv N$ $C\equiv O$	• Emission in silent region (1800–2800 cm^−1^) • Single narrow band • High encoding capability • Interference-free	• Early-stage development • Not commercially-available: self-design and synthesis requirement	
Protective shell Maintain the RR near the NP, stabilise the NPs and provide surface groups for bioconjugation	Hydrophilic short spacers Biomolecules Polymers Silica	Ethylene glycol Bovine serum albumin PEG Tetraethoxyorthosilicate (TEOS)	Electrostatic Interaction Covalent bond S–Au(Ag) interaction	• Improves colloidal stability • Terminal functional groups for bioconjugation • Core physical and chemical protection • Avoids loss of RR	• Increases SERS tag • Time-consuming • Labour-intensive	• Coating synthesis • Non-specific adsorption • Toxicity • Functional-end groups • Colloidal stability
Bioconjugation Molecular recognition of the target	Proteins: enzymes and antibodies Aptamers Small molecules	via COOH, NH_2_, SH click-chemistry	Covalent or non-covalent	• Specificity towards the target analyte	• Variable accordingly with chosen analyte	• Target-specificity ligand • Water solubility • Bioconjugation reproducibility • Price

To fulfil the promise that SERS tags hold, *i.e.* achieve specific and quantitative detection, care must be taken during the SERS-tags design. The choice of each component will ultimately depend upon the application and to combine all the necessary features without compromise is not a trivial task. For instance, in biomedical applications, one has to consider such aspects as the optical window of human tissue [68] and the pH of the medium, as well as phenomena such as tissue extravasation, opsonisation [83], cytotoxicity, enzymatic cleavage, cellular uptake mechanisms, the ability to avoid or resist the endolysosomal network, amongst others [15, 68].

### Signal amplifiers: inorganic nanoparticles

The main signal amplifiers developed for SERS tags are based on inorganic metal nanoparticles. Nevertheless, more recently, alternative materials such as graphene [84] and semi-conducting transition metal dichalcogenide substrates [85, 86] have been explored and show promising results as highly active SERS substrates.

The metal colloidal nanoparticle of SERS tags is essential for signal enhancement and to act as structural scaffold. Its optical properties, in particular the position of the LSPR band, will determine the efficiency. The LSPR of metal NPs strongly depends on the size, shape, and composition. Gold and silver are the metals more frequently used, gold being preferred in the biomedical field compared to other plasmonic NPs due to lower toxicity and high stability, whereas silver is favoured when the SERS efficiency is the limiting factor [87–89]. Alloys of Au and Ag combine the stability of gold and the enhancement activity of silver giving signals 10 to 100-fold higher than normal Raman signals. These types of alloys have been applied in SERS-based immunoassay [90], cell imaging [91], label-free chemical detection [92], and drug delivery [93].

Ever since Faraday’s work on AuNPs, numerous protocols have been developed for the synthesis of nanospheres of Ag and Au [94, 95]. Chemical synthesis by the reduction of metal salts is well-known and can be performed with a diversity of reducing and capping agents [96]. The capping agent works as a stabiliser to prevent the particles from aggregating, and the technique is a common and cost-effective approach to yield monodisperse metal spherical NPs of Ag and Au. The surface of the resultant Au and Ag NPs can be readily modified by stabilisers, thiols and disulphides providing a simple route to surface modification [97]. The importance of choosing an appropriate stabiliser is related to the further bio-functionalisation strategies [96].

Colloidal nanoparticle samples consist of a suspension of sub-micrometre sized metal particle (diameters usually between 3–200 nm) in a fluid. The narrow size distribution with little or no coalescence generates similar Raman scattering cross sections. Thus, individual spherical NPs give comparable SERS intensities, which is essential for quantification purposes [7]. These spherical NPs have long shelf lives with retained morphology which offers quality control for downstream applications [7]. However, the size range obtained for Au and Ag nanospheres synthesised by chemical methods is commonly of order 30–100 nm, resulting in a LSPR typically centred at 520 and 400 nm. Some protocols have been specifically designed to increase the size, to promote a LSPR red-shift, since it reduces fluorescence interference, but this approach is somewhat limited since increasing the size results in larger radiation damping effects which decrease the enhancement [98]. Oldenburg and co-workers circumvented this issue by demonstrating that it is possible to tune the LSPR across the NIR region by controlling the size of the silica core and the gold shell in core/shell nanoparticles [99]. Nevertheless, the sensitivity provided by a single nanosphere is low (EF ≈10^3^) and, to increase SERS tag brightness, the NP must possess hotspots. These locations at which the near field is enhanced occur at tight junctions between metallic NPs, and edges and vertices in non-spherical NPs [36, 37]. As a result, numerous different anisotropic NPs with intrinsic hotspots were explored (nanorods [100], nanostars [101], nanocubes [102], nanocages [103], nanotriangles [104], nanoprisms [104], clusters (see section 3.2), and others [105–108]). Nanorods (NRs) for instance, present a longitudinal and transverse LSPR band and are more efficient than nanospheres, with the efficiency being tunable according to the aspect ratio of NRs [100].

The advantage of using nanostars is that the sharp tips emanating from a core give several hotspots *per* particle [109, 110] with multiple resonances—“sharp tip effect”. Nanostars provide plasmonic near-field enhancements and lightning rod effect (maximised in a tip-to-tip nanostar dimer) which leads to EFs of orders of 10^9^ [7, 111]. Although challenging, their plasmonic properties can even be controlled by modulating the morphology of their tips and consequently enhancing the SERS signal [112]. Gold nanostars are typically produced by the seed-mediated approach [113] or by the 4-(2-hydroxyethyl)-1-piperazineethanesulfonic acid (HEPES)-based approach [111]. However, anisotropic etching can also be used to control the NP shape achieving new NP structures with high yield and purity that cannot be attained by the conventional chemical NP synthesis [114].

Note that this preference for NP with multiple hotspots at single particle level is only true in colloidal solutions. As demonstrated by Solís et al*.*, simpler morphologies might lead to higher enhancements when in an organised closed packed array [34]. Nevertheless, tips of nanostars and nanorods are dynamical evolving entities due to atomic migration and facet reorganisation and the modification of the tips, with time, can lead to the loss of SERS activity [115].

### Clusters

**Fig. 6:**
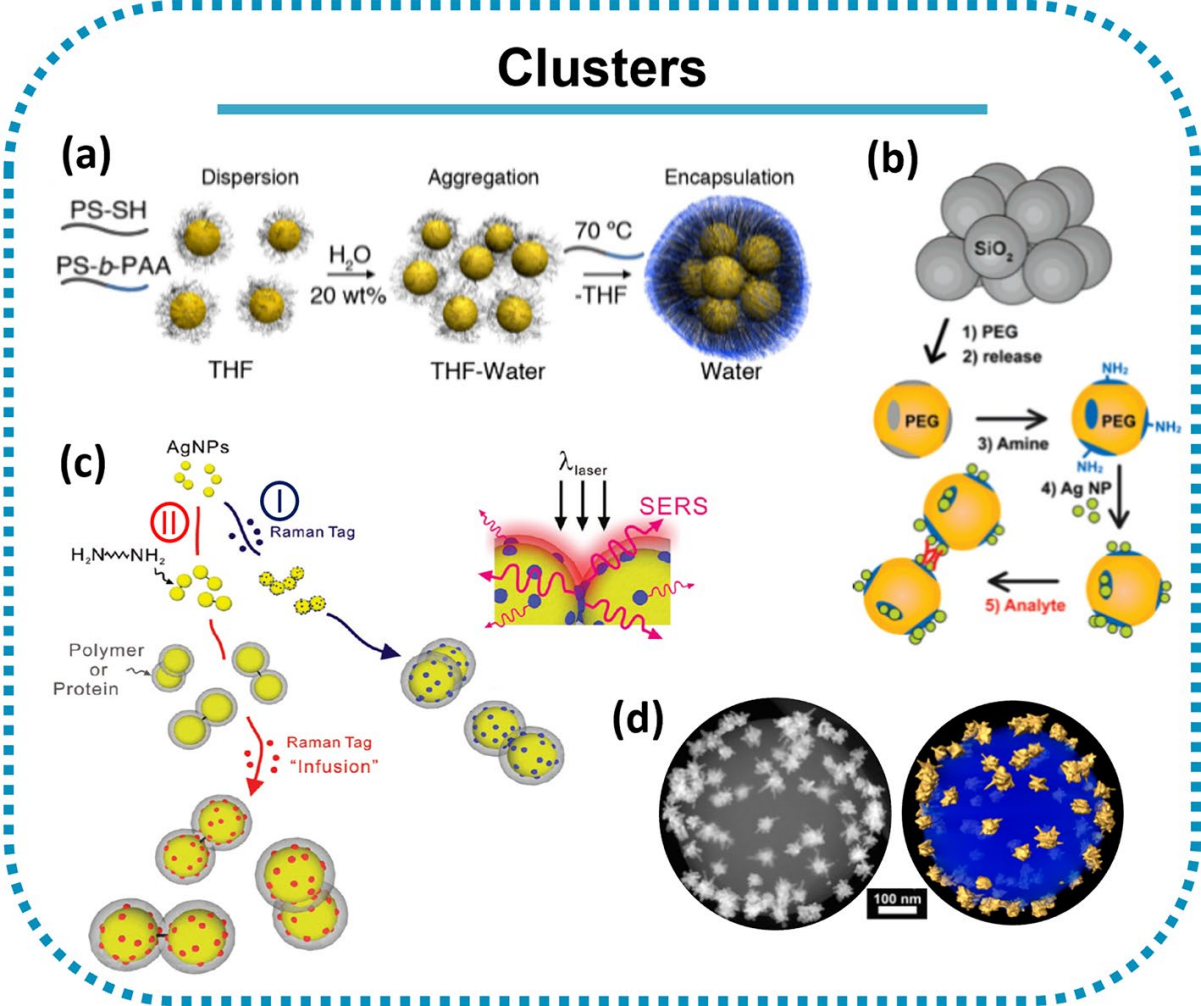
NP clusters. **a** Colloidal dispersion of polystyrene coated AuNPs in tetrahydrofuran (THF). Adapted from [118]. **b** Scheme of the process for molecular-linker-controlled NP-patterned microsphere assembly. Reprinted (adapted) with permission from [120]. Copyright 2007 American Chemical Society. **c** Scheme I. AgNPs are cross-linked with the bifunctional linker 4-aminobenzenethiol (blue) or 1,6-hexamethylenediamine (black), each then coated with a layer of polyvinylpyrrolidone-poly(acrylic acid). Scheme II. The SERS tag (red) is infused through the polymer coat. The inset represents SERS from tags in the junction. Reprinted (adapted) with permission from [122]. Copyright 2009 American Chemical Society. **d** Annular dark field-scanning-transmission electron microscopy (ADF-STEM) (left) and 3D reconstruction (right) images of a gold nanostar in a polystyrene bead. Reprinted (adapted) with permission from [124]. Copyright 2016 American Chemical Society

Nanoparticle aggregation also gives rise to hotspots in the regions between the NP surfaces, although the process is not well controlled and/or reproducible. To improve this, controlled NP assembly to form clusters (*e.g.* dimers [116] and core–satellite systems [117]) has been explored. Encapsulation of a few NPs to form dimer or trimer clusters was one of the first strategies explored [58]. The initial intention was to encapsulate single NPs but, due to the difficulty of controlling the number of NPs within the polymer, the approach was adapted to form clusters [58]. Notwithstanding this, although clustering promotes hotspot formation and thus the SERS signal strength, it still yields clusters of different sizes and thus irreproducible signals [58]. To ensure similar numbers of NPs in clusters, NP surface chemistry, ligand and solvent composition must be optimised. Grzelczak and collaborators circumvented this problem by adding a di-block copolymer containing a (hydrophobic) polystyrene block and a (hydrophilic) polyacrylic acid block (PS-b-PAA) (see Fig. 6a). As the water content is increased, the hydrophobic AuNPs slowly clustered and the addition of PS-b-PAA suppress further growth and stabilise the AuNP clusters [118].

Generating nanogaps has attracted much attention over the years and several approaches have been developed from a wide range of particles besides nanospheres, including nanowires, nanostars, among others [45, 58, 119, 120]. It is also possible to encapsulate single NPs and then promote their controlled aggregation, but this might not be a good approach to maximise SERS tag brightness since coating layers isolate the NPs, resulting in interparticle distances which are too large, even when coating layer molecular weight is tuned to try to minimise them [45, 58, 119, 120]. In some cases, the NPs are encapsulated first by a thin silica coating and then these NPs are used to form clusters through a second encapsulation [121] or a ligand (Fig. 6b) [122]. The thickness of the silica shell should be controlled to maximise SERS signal intensity. Shanthil and co-workers observed the shell thickness dependence in function of the enhanced Raman signal intensity being higher when the thickness was ≤ 10 nm. The observed enhancement at hotspots followed a 1/d^n^ dependence, which is in agreement with theoretical studies [38, 123]. To avoid increasing the SERS tag size, ammonium ions can be used to screen the negative charge on the silica coating as illustrated in Fig. 6c (scheme I), or a core–shell architecture may be adopted. In the latter example, the mesoporous silica between two layers of silver nanoparticles acts as a spacer regulating hotspots while simultaneously stabilising the NP (Fig. 6c—scheme II) [14].

Alternatively, as shown in Fig. 6d, NP clusters can be produced by depositing individual NPs (*e.g.* stars) on colloidal particle carriers, such as polymeric microbeads, with opposite charge enhancing the SERS signal and can thus be applied in bioimaging [124].

### Coding: Raman reporters

The RRs, also called Raman labels interchangeably in this work, are molecules responsible for the indirect detection of a selected analyte – extrinsic labelling. The enhancement observed in SERS requires that RR molecules be bound to or near the surface of the metal, since the EM enhancement is distance-dependent and CHEM requires chemical bonding [8]. An ideal RR exhibits the following properties: (i) high Raman scattering cross sections for high brightness; (ii) a small number of atoms and/or high symmetry, thus presenting a limited number of vibrational bands to reduce possible overlaps and improve spectral multiplexing; (iii) minimal photobleaching for signal stability upon laser illumination, and (iv) ability to bind to a metal surface usually by nitrogen- and/or sulphur- containing molecules for chemisorption onto the metal surface. If the RR does not contain any of these surface-seeking groups, it should still be strong enough to prevent desorption during the following modification steps [7, 12, 13, 63, 77].

**Fig. 7:**
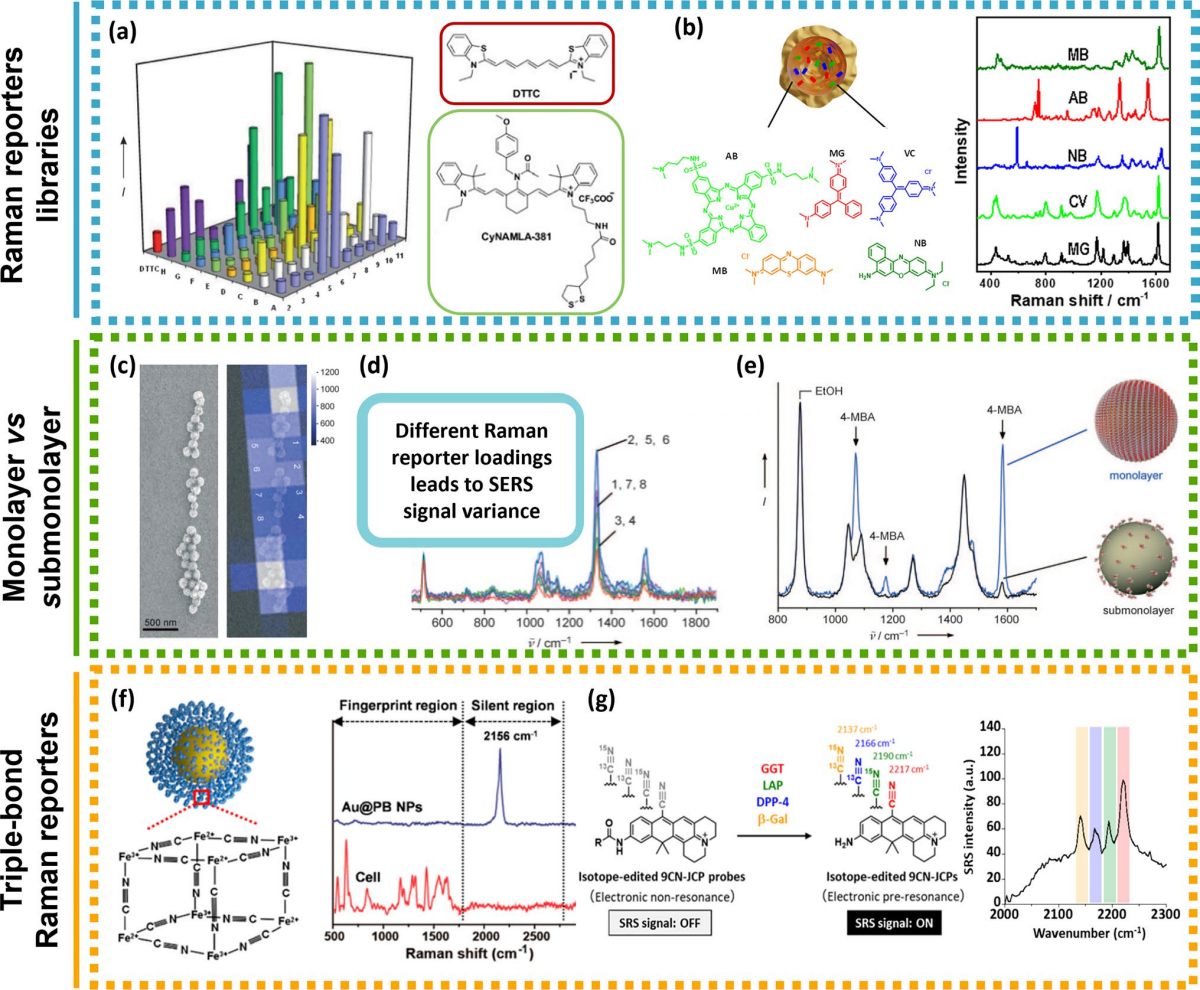
RR libraries examples. **a** Comparative SERS intensities with excitation at 785 nm of the whole 80-member of the CyNAMLA library. DTTC was used as the reference and is plotted as a red bar and the molecular structure is represented in the red box. The highest NIR SERS intensity was obtained for the compound CyNAMLA-381 (green bar) and its molecular structure is represented in the green box. Adapted from [125]. **b** Plasmonic nanocapsule encoded with five different Raman reporters. Adapted from [142]. RR coverage influence. **c** Left: scanning electron microscopy (SEM) image of silica-encapsulated SERS reporters on a silicon wafer. Right: False-colour SERS map overlaid with the SEM image. **d** SERS spectra of 5,5’-dithiobis(2-nitrobenzoic acid) obtained from squares labelled 1–8 in (**c**). **e** Impact on SERS signal strength due to differences in surface coverage: complete SAM produces a stronger SERS signal compared to submonolayer coverage with RRs. Triple-bond RRs [128]. **f** Left: Schematic illustration of Prussian blue (PB) coated AuNPs. Right: Raman spectra of PB coated AuNPs and HepG2 cells. Biological species exhibit complex multiple bands in the fingerprint region whereas the AuNPs-PB possess an intense and sharp single band (2156 cm.^−1^) in the cellular Raman-silent region throughout the whole spectrum. Reprinted (adapted) with permission from [136]. Copyright 2017 American Chemical Society. **g** Left: Development of activatable Raman probes for enzyme activities based on isotope-edited xanthene derivatives bearing a nitrile group at position 9 (9CN-JCPs). GGP: γ-glutamyl transpeptidase; LAP: leucine aminopeptidase; DPP-4: dipeptidyl peptidase-4; β-Gal: β-galactosidase. Right: stimulated Raman scattering spectra of enzyme activities obtained from H226 cells. Adapted from [133]

In terms of RRs there are two main options, organic chromophores or small aromatic thiols. Chromophores are excellent RRs since, under resonant Raman conditions (SERRS), the enhancement can increase 10–100-fold compared to conventional SERS experiments with electronically non-resonant molecules. Although promising for multiplexing, combinations of different dyes is difficult to accomplish, due to the lack of dyes with similar absorption bands or with a broad range of excitation wavelengths. Efforts have been made to synthesise reporters through synthesis and screening members of libraries of compounds such as triphenylmethine and tricarbocyanine [125, 126]. For instance, synthesis of an 80-member tricarbocyanine library allowed the identification of CyNAMLA-381 as a NIR SERS reporter with 12-fold higher sensitivity than the standard 3,3’-diethyl-thiatricarbocyanine (model reporter for NIR excitation) (Fig. 7a) [125]. Still, this has proven a difficult task and the examples in literature are scarce [125]. In case of fluorophores, the tag design is especially important, since the fluorescence can generate an intense background [58].

Aromatic thiols, isothiocyanates and amines on the other hand, are small, also have high Raman cross sections, and are poorly fluorescent. Due to their structure that contains surface-seeking moieties for chemisorption, they can form a self-assembled monolayer (SAM) on gold surfaces *e.g. *via Au–S bonds [127]. The dense packing in SAM offers a uniform orientation of RR molecules and therefore the response to an individual binding event is markedly amplified. Consequently, other molecules present in the surrounding medium, from buffers or synthesis reagents, are not adsorbed on the metal surface and spectral interferences are minimised, generating maximum SERS brightness with reproducible SERS signatures. A study done by Küstner et al*.* [128], showed a 22 times increase of mercaptobenzoic acid (MBA) SERS signal when the NPs had a complete monolayer coverage, compared to those NPs with sub-monolayer coverage (Fig. 7c, d and e). This confirms what is illustrated by Eq. 1, that the SERS brightness of a single tag is proportional with the number of RRs attached to the NP:

$${P}^{SERS}\left({v}_{s}\right)=N{\sigma }_{effective}^{SERS}\bullet I\left({v}_{L}\right)\Leftrightarrow$$

1 $${\Leftrightarrow P}^{SERS}\left({v}_{s}\right)=N{\sigma }_{adsorbed}^{R}{\bullet \left|A\left({v}_{L}\right)\right|}^{2}\bullet {\left|A\left({v}_{s}\right)\right|}^{2}\bullet I({v}_{L})$$

The SERS brightness *i.e.* SERS Stokes signal ${P}^{SERS}\left({v}_{s}\right)$, depends on excitation laser intensity $I({v}_{L})$ and on the effective SERS cross section ${\sigma }_{effective }^{SERS}= {\sigma }_{adsorbed}^{R}{\bullet \left|A\left({v}_{L}\right)\right|}^{2}\bullet {\left|A\left({v}_{s}\right)\right|}^{2}$, which is influenced by the Raman scattering cross section of the adsorbed molecule ${\sigma }_{adsorbed}^{R}$, that is enhanced due to CHEM when compared to the cross section in a “normal” Raman experiment ${\sigma }^{R}$, and $A({\nu }_{L})$ and $A({\nu }_{S})$ are laser and Raman scattering field enhancement factors, respectively. $N$ is the number of molecules that undergo the SERS process [80, 89]. Therefore, many reports try to increase the number of labelling reporters to increase the optical quality of SERS tags, the formation of SAM being the ultimate optimisation. A combination of SAM with the well-known chemistry of aromatic thiols makes them suitable to quantitative estimations of SERS EFs which provides multicoded tags for multiplexing analysis [129, 130].

However, SAM of RRs is limited to a few charged examples [128], for instance MBA, which has a thiol group for Au attachment and carboxyl group to generate negative charge in solution. It is difficult to produce these high coverage densities of uncharged molecules on NPs without inducing severe aggregation.

Instead of selecting the suitable RRs and optimising the functionalisation according to each chemistry, some researchers use custom-made RRs [126, 131–134]. For example, Graham et al*.* were able to perform multiplexed experiments for DNA detection with a range of benzotriazole azo dyes which are excellent for SERRS [132]. Other groups used dyes with alkynes (C$\equiv$C) [135], nitriles (C$\equiv$N) [136, 137], azides (N_3_) [138], and deuterium (C–D) [139] that show single narrow bands in the biological Raman-silent spectral window (1800–2800 cm^−1^), avoiding background interference [134, 137] (Fig. 7f). These triple-bond-conjugated dyes are generated from a suitable scaffold dye and create palette of NIR dyes able for multiplex detection [131, 134, 137] or even for protein screening, allowing identification of drug binding sites [140]. Nevertheless, alkyne RRs biomolecule application is limited, due to their small Raman scattering cross-section. Chen et al*.* surpassed this limitation by combining 4-ethylbenzenethiol (EBT) and alkynes [57]. These EBT derivatives were able to generate unique and high SERS signals within the cell-silent window. As a result, three-colour SERS imaging of a cell was achieved from this alkene SERS palette. A remarkable approach to explore alkyne or nitrile tags in multiplexed imaging was demonstrated by Fujioka and co-workers [133]. They were able to synthesise Raman labels that generate strong signals due to electronic preresonance upon reaction with enzymes (Fig. 7g). Thus, the RR signal was manipulated to be exhibited during imaging detection of enzymatic activity in live cells [133]. Interestingly, some other RR properties might be explored, besides their inherent high Raman scattering cross sections. Bando and co-workers, for instance, used MBA functionalised Ag nano-assemblies to monitor in 3D the variation of the intercellular surrounding pH with spatial accuracy of several tens of nanometres and a temporal resolution of 200 ms [141]. These cases illustrate the need for rational design and screening of novel reporters.

### Protection and stabilisation: coating layer

At this stage, the strong and specific SERS signal from the RR is assured by the proximity to the plasmonic NP. To enclose the NP and the RR, a coating is usually employed [13]. To guarantee the brightness stability of SERS tag, the interaction between NP and the RR should be much stronger than that between the NP and the coating protecting layer, to avoid RR displacement. This protective shell avoids the displacement of RRs from the proximity of the metal surface as well as adsorption of spectrally interfering molecules. Also, the encapsulant improves the stability of the colloidal particles, making them suitable for dispersion in biological fluids without being prone to aggregation in high-ionic-strength media. Furthermore, the coating improves water solubility and biocompatibility, since it prevents exposure of the biological environment to the metal substrate [7, 8, 12, 13, 15, 63, 82].

Simultaneously, coating can provide a surface for subsequent bioconjugation. Biofunctional stabilisers for instance are used to stabilise and introduce chemical functionalities (*e.g.* thiolated-PEG molecules having carboxylic, amine, azide groups, etc.). A highly important function of the coating shell is to prevent non-specific binding, *i.e.,* the binding selectivity should only occur by the target-specific binding molecule [12, 13, 15].

The coating typically falls into four classes: (i) short spacers that provide direct hydrophilic stabilisation of SAM; (ii) biomolecules, (iii) polymers, (*e.g.* PEG), and (iv) glasses such as silica.

**Fig. 8:**
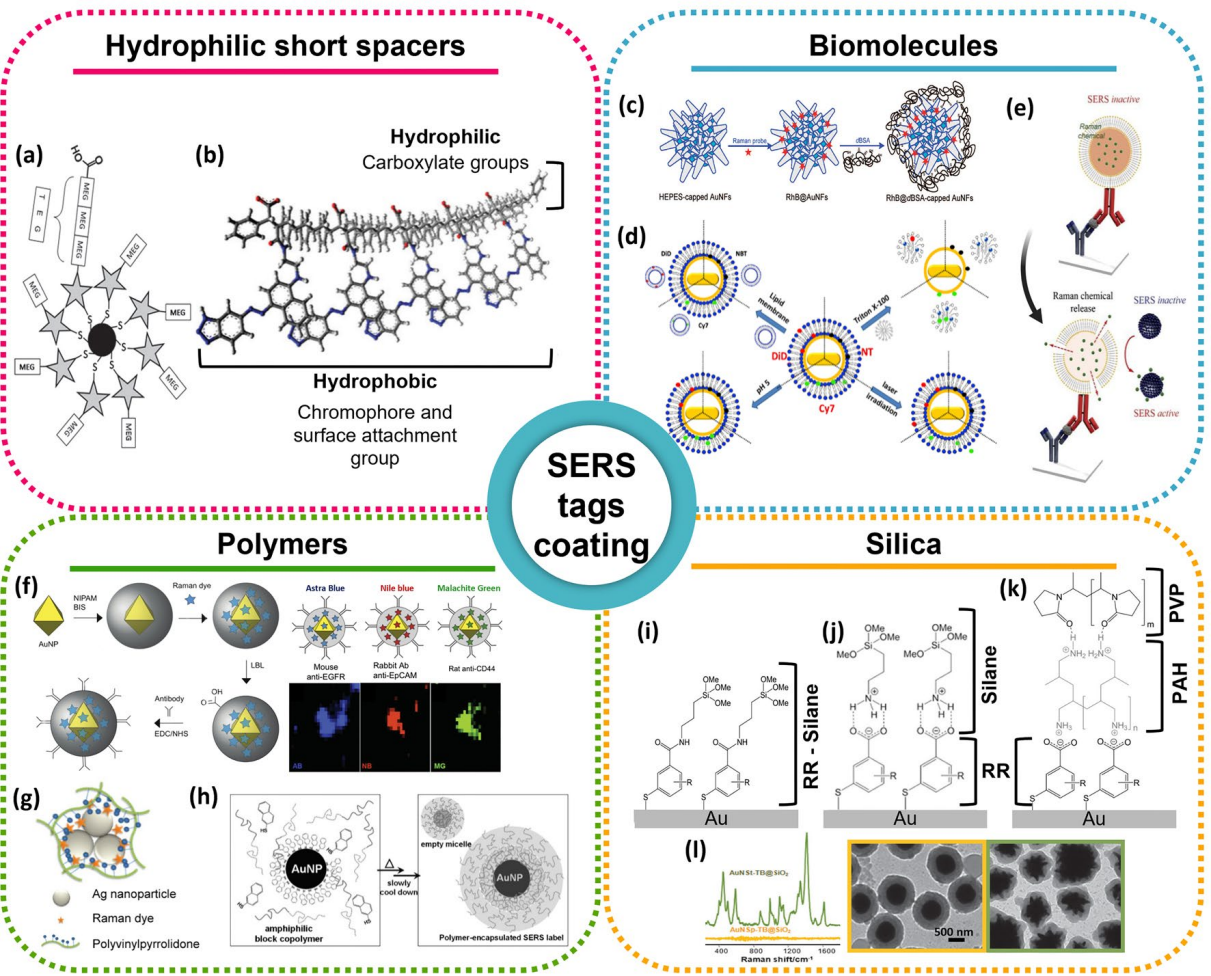
SERS tags coating approaches. Hydrophilic short spacers: **a** mono- and triethylene glycols are conjugated with the RRs to stabilise the SAM and provide terminal carboxy moieties to bioconjugation of biomolecules. Adapted from [143]; **b** scheme of the chemical structure of the polymer dyes. Adapted from [145]. Biomolecules: **c** denature BSA protected gold nanoflowers as SERS tags. Reprinted (adapted) with permission from [108]. Copyright 2008 American Chemical Society; **d** location of each RR in lipid-coated SERS tags and the lipid dissolution after several physicochemical signals. Reprinted (adapted) with permission from [149]. Copyright 2016 American Chemical Society. **e** RR-encapsulated liposome-that upon stimulus release the RR and a SERS signal is generated when in contact with Au@Ag-assembled silica NPs [90]; Polymers: **f** Left: Au@pNIPAM SERRS-encoded tags. Right: SERRS tags scheme and corresponding map using different bands for each reporter. Adapted from [162]; **g** PVP-coated, Raman-tagged AgNPs. The aggregation is induced by adding a NaCl solution and the addition of PVP forms a shell around the SERS tags. Adapted from [157]; (**h**) Polymer-encapsulated AuNPs by self-assembly by heating the AuNPs with 2-naphthalenethiol and PS154-b-PAA60 followed by gradual cooling. Adapted from [150]; Silica: Encapsulation routes for silica coating: **i** RR molecules with covalently bound terminal SiO_2_ precursors; **j** silane as a noncovalently bound SiO_2_ precursor bound to the RR molecule with a polar head group and **k** use of two polyelectrolytes (PAH and PVP) to render the surface vitreophilic. Adapted from [121]. **l** Left: SERS spectra of AuNSp-TB@SiO_2_ and AuNSt-TB@SiO_2_ SERS tags; Right: TEM images of each SERS tag. Adapted from [160]

Using a mixture of the hydrophilic spacers monoelthylene glycol (MEG) and triethylene glycol (TEG) with different terminal groups (OH and COOH, respectively), not only increases the stability towards physiologically relevant conditions, but also provides steric accessibility of the SAM for bioconjugation via the longer TEG-COOH spacer (Fig. 8a) [143, 144]. Other examples include functional SERRS labels such as multidentate macromolecules with regions with different degrees of hydrophilicity (Fig. 8b) [145]. Nevertheless, requirement for significantly longer EG chains and the synthesis complexity degree impairs its widespread use, especially for in vivo applications.

Coatings of biological nature are attractive due to provide stability while preserving biocompatibility. Similarly to EG spacers, biomolecule coatings might be limited to detection in vitro or ex vivo [146, 147], due to the dynamic evolution of NP-protein corona and enzymatic cleavage when in contact with biological environment [148]. Denatured bovine serum albumin (dBSA), for instance, offers 35 cysteine residues in each BSA molecule able to protect gold nanoflowers with embedded Raman probes (Fig. 8c) [108]. Another group of biomolecules used as coating are the phospholipids. Liposomes are self-assembled into organised structures that truly mimic cell membranes and thus render NPs biologically compatible and highly versatile. As Su and colleagues [149] discovered, RRs are capable of exhibiting a stimulus responsive SERS signal dependence when interacting with NPs and lipid layers (Fig. 8d). In fact, Pham et al*.* used liposomes to enhance the response from the encapsulated RR when a positive detection from the SERS-based immunoassay was accomplished (Fig. 8e) [90].

One of the most commonly used protective layers against NP aggregation and prevention of the loss of the RR are polymers [150]. PEG is a linear polymer that when anchored to NPs surfaces, PEG preserves enough space to bind the RR while precludes the binding of other molecules by steric effects. Nevertheless, the batch-to-batch variability, even between commercial brands, and the high price of thiolated PEG molecules hinders the ability to perform large-scale synthesis of bioconjugates [151].

The encapsulation of NPs has also been attempted by amphiphilic polymers [152], polymeric matrices [153] and smart polymer-based composites [154]. Polymers such as poly(N-isopropylacrylamide) (pNIPAM) have proven to stabilise the NPs and to have a low Raman background, which is essential in ultrasensitive applications [155]. Thanks to their porous nature, the RRs can be incorporated through diffusion and trapped in a sealing porous shell by layer-by-layer (LbL) polyelectrolyte coating which offers a reactive surface for covalent conjugation with antibodies (Fig. 8f) [155]. In order to form controlled clusters, polyvinylpyrrolidone (PVP), which is usually used to control shape in the polyol process and to favour steric stabilisation of noble metal NPs [156], can be used to manipulate the aggregation degree of labelled AgNPs (Fig. 8g) [157]. Furthermore, polymers have the ability to be manipulated to respond to an external stimulus, either endogenous (e.g. pH, glutathione, reactive oxygen species, enzyme, hypoxia) or exogenous (e.g. light, temperature, ultrasound, magnetism and electric field), or even a mixture (Fig. 8h) [154].

Silica encapsulation to protect SERS tags allows to combine SERS signal from single metal NPs with the robustness offered by glass encapsulation (Fig. 8i) [78, 158]. To avoid competition between RRs and silane groups and low SERS brightness from the sub-monolayer coverage, Schütz et al*.* used LbL deposition of poly(allylamine hydrochloride) (PAH) and PVP creating polyelectrolyte-coated SAMs and finally glass encapsulation using tetraethoxyorthosilicate (TEOS) (Fig. 8j and k). Hence, the deposition of a silica shell has been used extensively over a wide number of NPs shapes such as NRs [159], NP dimers or trimers [69], nanostars [160] (Fig. 8l) and assemblies [141]. Nevertheless, the several steps required, in different solvents and multiple centrifugations makes the approach labour-intensive and time-consuming. To avoid nonspecific binding, an outer layer of PEG might be inserted which increases the coating layer and thus the SERS tag size which potentially leads to steric hindrance and lower SERS signals [161].

### Specificity: target-specific ligands

The specificity towards the target analyte is provided by the last step of the preparation of SERS tags, the bioconjugation with a target-specific entity. The most frequently used biomolecules for targeting molecules are antibodies [163], aptamers [79], oligonucleotides [164], and small ligands such as folic acid [165, 166], and toxins [167].

Antibodies and their fragments are among the proteins with the widest applications in SERS tags because the antigen–antibody region is highly specific [168]. However, the success of antibody-antigen interaction depends on epitope presentation and binding chemistry [168]. A recent study by Avvakumova et al*.* showed how the selection of the conjugation strategy, including antibody orientation or the presence of a polymeric spacer or recombinant protein linker, has impacts on targeting cancer cells [163]. Also, in the presence of high complexity samples, antibodies might exhibit cross-reactivity, especially if they are polyclonal. Other disadvantages are related to high cost and long-term stability [169].

Aptamers are gaining prominence as replacement for antibodies in some applications, since they can bind with equal, if not higher, selectivity. Aptamers are short strands of oligonucleotides (15–40 bases) that form complex three-dimensional structures with high binding affinities [170]. Their advantages include minimal immunogenicity and higher resistance to degradation, under physiological conditions, compared to antibodies. Additionally, they are designed and selected by systematic evolution of ligands by exponential enrichment (SELEX) which represents an easier and lower cost synthesis than antibodies [170]. Conversely, aptamers are still quite limited in the possible number of targets and only to soluble proteins, which are morphologically different from their membrane-embedded counterparts [170]. A very recent review focused on the working strategies of aptamer-based SERS biosensors, highlighting the advantages and perspectives [79].

A smaller and more stable alternative to proteins are molecules such as folic acid (FA), a B vitamin type necessary for amino acid and nucleic acid synthesis inside cells [165, 166]. The overexpression of FA binding proteins has been successfully explored in discriminating between cancer and non-cancer cells. Thus, ATP-AuNPs conjugated with FA exhibit the capability of single-cell cancer screening and by replacing FA by FA competitors (*e.g.* aminopterin and methotrexate) it is even possible to quantify the amount of drug delivered to the cells [165, 166]. Toxins that interact with cellular metabolism have also been explored in detection systems. Zhang et al*.* produced a nanobeacon composed of AuNPs with a phospholipid bilayer coating with embedded RRs and a binding ligand with high-affinity to cholera toxin [167]. Molecules of the family known as pyrrole − imidazole polyamides enable binding within the minor groove of dsDNA and consequently can be exploited to cause AuNP aggregation in the presence of one- and two-base-pair mismatches from fully complementary dsDNA sequences relative to dsDNA sequences [171]. However, these kind of nanosystems lack universality, with applications being limited to dsDNA detection.

Parameters such as purity, affinity, and orientation of these elements attached to the NPs influence greatly the targeting efficiency. The bioconjugation strategy depends on the configuration of the SERS tag components chosen, up to this point, which influence the stability and performance of the SERS tag. The target moiety needs to be bound in a way that ensures no exchange with other tags and the coverage should be optimised to maximise the amount of target-ligand interaction while minimising nonspecific interactions. Covalent bonds are preferred since they do not allow “cross-talk” between different SERS tags. Conjugation can be done by direct conjugation to unprotected SERS tags, or through the conjugation of ligands to protected SERS tags.

For unprotected SERS tags, the adsorption of the targeting entity can be performed by electrostatic interactions or covalent binding. Noncovalent methods might lead to a co-adsorption of a thiolated RR and antibody molecules leading to a mixed monolayer [172]. If the thiolated RR with a functional terminal group forms a SAM, then the protein can be covalently attached through the formation of an amide linkage [14]. In this second approach, Ni et al*.* circumvented non-specific binding and “cross-talk” between different SERS reporters but failed to avoid the steric accessibility of COOH moieties in the SAM on the Au surface for posterior bioconjugation [14, 172]. A further improvement was developed by Porter and co-workers that entailed introducing mixed thiols, one aromatic thiol for the characteristic Raman signal and a second alkylthiol with a terminal functional group (*e.g.*, succinimidyl group) for bioconjugation [14].

SERS tags can also include oligonucleotides bound directly to the NP for DNA detection. Graham and McKenzie, for instance, conjugated thiolated DNA to SERRS labels through the strong Au–S bond for DNA detection [164]. Incubation of thiolated aptamers or oligonucleotides can partially replace the stabilising molecules from NPs (such as citrate) and still maintain colloidal stability.

When the SERS tag is protected by a stabilising shell, the terminal functional groups of the protective entity most frequently end in carboxyl groups or primary amines. These groups can then form an amide bond by carbodiimide activation [173]. This type of coupling strategy has been employed to hydrophilically stabilised SAMs, polymer- and silica-encapsulated SERS nanoprobes [173]. Other chemical approaches include binding of biotin-modified tags to avidin or streptavidin-modified NPs, silane chemistry, Michael addition, click chemistry, or Diels − Alder reaction, among others [174].

The SERS tag signal generation is controlled by the interaction of recognition biomolecules with the specific target. Consequently, parameters used in the chosen conjugation scheme, namely, chemical reactions involved, linker, anchoring groups, stoichiometry between biomolecules and NPs, and conjugation conditions, determine the effectiveness of the SERS tag and should be designed accordingly for the given target and assay. Due to its importance, the following section is dedicated to bioconjugation strategies.

## Bioconjugation strategies

Bioconjugates in nanotechnology are a popular approach that have two major biomedical applications, namely therapy and diagnosis. They consist of a hybrid material involving the attachment of inorganic NPs to biomolecules allowing the NPs to interact with biological systems in a specific manner [96, 175]– [177]. Nanoparticle-biomolecule conjugates combine the beneficial properties of inorganic particles such as magnetic moment or SPR, with biorecognition provided by proteins (*e.g.* enzymes or antibodies), or nucleic acids such as aptamers or complementary oligonucleotides [96, 151, 178, 179].

Depending on the application, it is necessary to choose the targeting component and the strategy to attach it to the surface of the particle considering the kinetics of the reaction and the stability during the assay. Assuring that the NPs remains stable in solution is often the biggest challenge while bioconjugation process takes place. This is a consequence from using compounds for bioconjugation that disturb the fragile balance between attractive and repulsive forces. Besides colloidal stability, bioconjugation processes can have significant impacts on physical–chemical properties such as size, surface charge, hydrophobicity and targeting features.

Due to the diversity of NPs and biomolecules reporter so far, the choice of a coupling strategy is nontrivial. Depending on the nanosystem, different functional groups, biomolecules to attach, stability and hydrophobicity degrees, will dictate the bioconjugation conditions (*e.g.,* pH, temperature, ionic strength, solvent choice, structure of the surfactant). As a result, there are no standardised protocols for NP bioconjugation, and each research group must balance pros and cons.

**Fig. 9:**
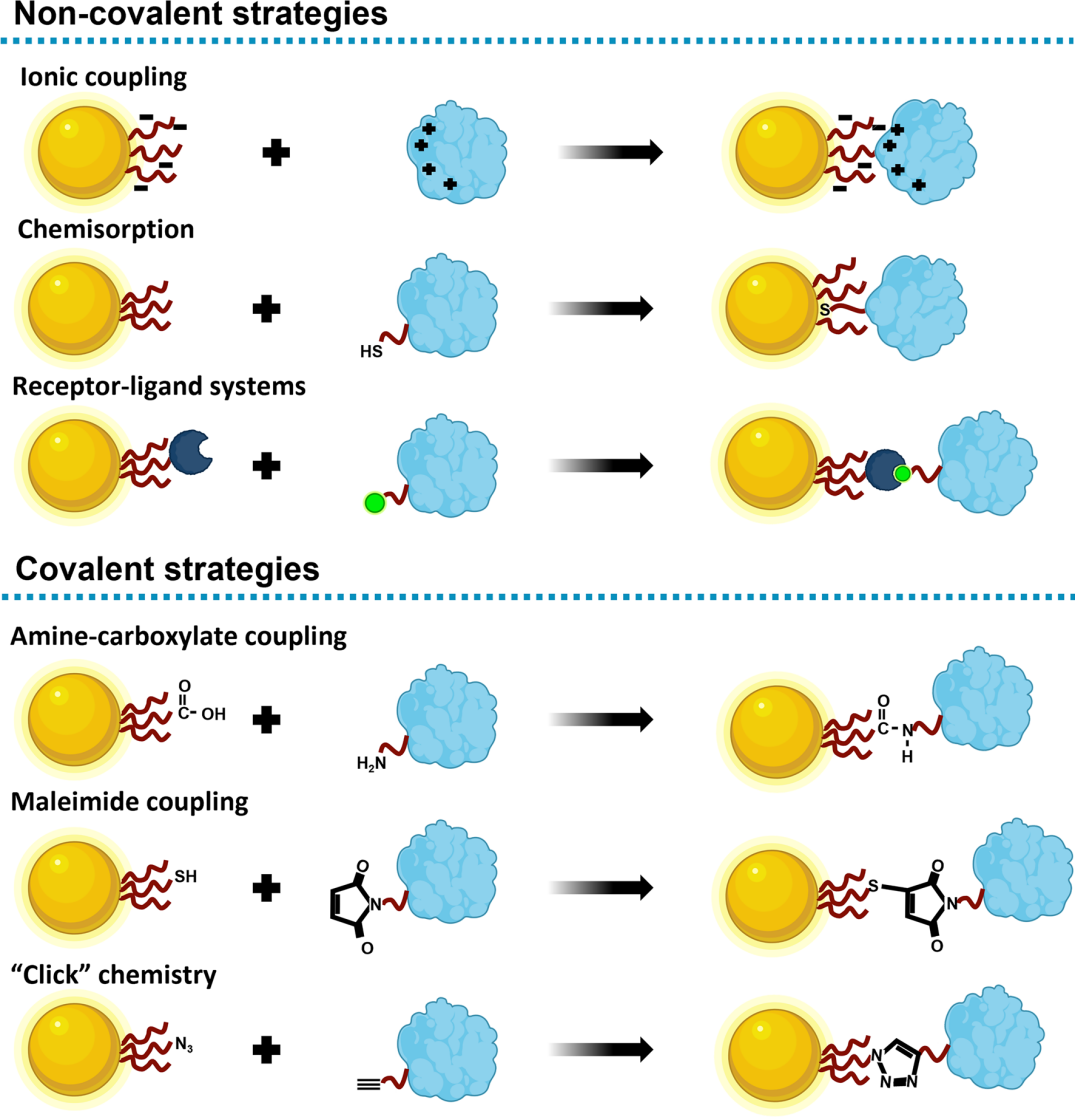
Conjugation strategies for achieving NP-biomolecules conjugates. Non-covalent strategies: ionic coupling, chemisorption (mainly accomplished by the affinity of sulphur atoms towards gold surface), and receptor-ligands systems such as avidin/biotin interaction with a monomer of biotin. Covalent strategies: covalent attachment to a ligand using amino-carboxylate coupling, maleimide coupling known as Michael addition, and “click” chemistry reaction using an azide tagged NP and an alkyne tagged protein

Immobilisation of biomolecules can be accomplished by two main mechanisms, simple adsorption, or chemical linkages. Adsorption is very useful because takes profit from the non-covalent forces (hydrogen bonding, ionic interactions, and Van der Waal forces). Chemical linkages allow to immobilise the biomolecules on a biocompatible matrix normally using bifunctional linkers. The strategies for the conjugation of biomolecules to NPs and a comprehensive comparison between covalent and non-covalent conjugation strategies is summarised in Table 3 and a few bioconjugation strategies are represented in Fig. 9.

**Table 3:** Comparison of the covalent and non-covalent conjugation strategies in bioconjugation processes and possible interactions between biomolecules and nanoparticle’s surface in bioconjugation process [96, 176, 180–182]

Non-covalent interactions	Advantages	Disadvantages
*Spontaneous absorption of biomolecules onto the surface of stabilised NPs*	• Convenient and simple • Do not require any additional chemical components • Most useful in understanding physicochemical interactions at the nano–bio interface • Used to reduce non-specific reactions and aggregation • Affinity-based receptor-ligand systems provide strong bonds with high binding affinity to cells and resistance to: o pH; o Temperature variations; o Denaturants	• Random orientation of proteins reducing its activity • Necessity of a high concentration of biomolecules for the preparation of biomolecule–gold particle conjugates • Difficult control over biological response due to the bioconjugate is mainly formed by electrostatic attractions • The binding is greatly influenced by changes in: o pH; o Ionic strength *e.g.*, increasing the electrolyte concentration shields the attractive electrostatic interaction causing the desorption of biomolecules • Possible displacement by other molecules on the NPs surface specially in complex biological samples • Washing buffers can remove loosely bound proteins and reduce non-specific interactions
Electrostatic interactions Positively charged groups in biomolecules are attracted by the negative charged surface of the metal NPs or vice versa		
Hydrophobic interactions Attraction between hydrophobic parts of the biomolecule and the metal nanoparticle surface.		
Chemisorption Donation of unshared electron pairs from free atoms of the biomolecule to the metal conducting electrons (*e.g.* sulphur-gold)		
Adaptive molecules Affinity-based receptor-ligand systems such as streptavidin–biotin		

Covalent interactions	Advantages	Disadvantages
*Chemical bond between biomolecules and the surface of NPs*	• Thermally stable (desirable in applications that require thermo cycling) • Allows 10–40% more protein via covalent • Prevents elution of bound protein increasing the stability • Correct spatial biomolecule orientation can be difficult via physical adsorption, whilst covalent attachment can orient the molecule properly, yielding increased activity and lower reagent consumption	• Effectiveness limitations when release is required as in drug release systems • Attachment of highly active antibodies can be impaired due to pH requirement for successful cross-linking reaction • Depending on the strategy, covalent binding can originate random orientation of proteins reducing its biomolecule activity • Covalent attachment can force the protein to have unfavourable interactions with the NP surface coating ligand, causing unfolding
Conjugation chemistry Exploiting functional groups on both particles and biomolecules such as bifunctional linkers of mediator linkers		
Maleimide coupling Diels–Alder cycloadditions with dienes; 1,3-dipolar cycloadditions with nitrones and azides; Michael-type additions with thiols and amines		
Click chemistry Refers to a family of reactions that are modular, stereospecific, and high yielding

### Characterisation of bioconjugation process

The choice of bioconjugation strategies to efficiently design bioconjugates will depend on the application. Regardless of the method, is important that the conjugation is specific for the biomolecule to bind to the NP, while not interacting with its native structure and potentially altering its biological activity. Ideally, the conjugation strategy results in a stable colloidal system provided by the attachment of biomolecules in a controlled manner, whereby the concentration is sufficient to cover the NP surface and maintain its activity. This is not always trivial, as parameters such as pH varied during bioconjugation can greatly influence the tertiary structure of proteins in the bioconjugates [183].

Once biomolecules are conjugated to NPs, their properties can change, depending on their microenvironment on the NP surface. Conjugated proteins, for instance, are exposed to neighbouring surface ligands and the metallic surface which influences the orientation or the secondary structure of proteins and ultimately perturb their function. For this reason, understanding the supramolecular interactions between proteins and NPs is crucial to the use of the bioconjugates in sensing and therapeutic applications. The nano-bio interface is a combination of three dynamically interacting components: (i) the nanoparticle surface, the characteristics of which are determined by its physicochemical composition; (ii) the solid–liquid interface and the changes that occur when the particle interacts with components in the surrounding medium; (iii) the solid–liquid interface’s contact zone with biological substrates [174, 177, 183–186].

When NPs come in contact with a biomolecule solution, a NP-biomolecule interface is determined by a range of equilibrium constants representing the different (and competitive) binding mechanisms that can result in structural rearrangements.

Considering the amount of biomolecule adsorbed as a function of the amount of biomolecule in solution, a Langmuir isotherm can be obtained [187]. When in low biomolecule concentrations, a linear response is found whose slope is an indication of the binding affinity (given by the equilibrium constant K), slowly reaching a plateau of binding at high biomolecule concentrations indicating saturation of the surface [187]. Parameters such as hydrophobicity, steric hindrance, size, morphology and curvature of the NPs influence the amount of entities bound to the NP [174, 177, 183–186, 188]. This nanoparticle-biomolecule corona is not at equilibrium but in dynamic evolution, such that the proteins and surface coating ligands on the NP can exchange on and off the surface. The initial stage is characterised by a rapid adsorption of biomolecules onto the NP surface. As time passes (hours to days), the biomolecules undergo structural rearrangements such as unfolding. The surface properties of the NP may lead to epitope exposure of proteins leading to different binding efficiencies and ultimately have a strong impact on the final application. Likewise, biomolecule adsorption can cause secondary effects such as surface reconstructions of the NP, changing the surface energetics, dissolution of the NP material, among others [184].

Hence, the biophysical properties of the bioconjugate can be altered and the function of the attached biomolecules impaired. There have been investigations to determine the dominant effects that modulate protein function [189]. One of the main difficulties is the limitation of techniques available to quantify the interface properties and weak interactions. Techniques such as fluorescence resonance energy transfer (FRET), isothermal titration calorimetry (ITC), fluorescence activated cell sorting (FACS), SPR spectroscopy, and ELISA can characterise stronger interactions but overlook the weak interactions that are characteristic of nonspecific adsorption and predominantly comprise bioconjugates [189]. Others, such as scanning tunnelling microscopy (STM), X-ray diffraction (XRD), low energy electron diffraction (LEED), X-ray absorption (XAS), X-ray photoelectron spectroscopy (XPS), are extremely useful to analyse inorganic NPs, but not biological molecules, due to their dependence on ultrahigh vacuum conditions, absence of water or low temperature [189]. Examples of techniques that probe the biophysical properties of the nanoparticle-biomolecule as a whole and have been used to study how a structure of a biomolecule is influenced by the NP surface chemistry, material, size via curvature effects and conjugation chemistry [189], include analytical ultracentrifugation (AUC), circular dichroism (CD) spectroscopy, FRET, mass spectrometry, and gel electrophoresis [185]. For instance, conformation changes upon bioconjugation can be easily accessed by spectral deconvolution of CD spectra, which, for example demonstrated a decrease of 16% in α-helical content of Cc when the protein was conjugated to NPs coated with the negatively charged ligand bis-(p-sulfonatopehnyl) phenylphosphine, and 28% when the positively charged ligand, aminoethanethiol was used [181]. Changing the labelling site for attachment to the NP, showed that linking the NP to a residue near the N–C foldon regions resulted in partial unfolding of Cc. These studies serve as a guide for developing design rules for bioconjugate formation which could lead to the development of more effective bioconjugates.

## SERS-based microfluidic biosensors

Microfluidics has become essential in the development of POCT systems [190–192]. For a drop of blood, microfluidics can combine sample collection, pre-treatment, on-chip filtration, reagent mixing, signal detection, and readout into a single device, in other words, integrate all molecular diagnostic laboratory methods in one chip, hence the term "lab-on-a-chip" [24, 193].

Since their invention, microfluidics systems have attracted attention in a variety of biodiagnostic methods including PCR [194], LAMP [195], chemiluminescence-based assays [196], and immunosensors [24, 197–200] platforms. Microfluidic immunosensors can be much more efficient and rapid than conventional immunoassay methods, due to the increase of surface-to-volume ratio that maximises mass transport in immunoreactions which, in turn, leads to a more rapid analysis [24, 201]. Moreover, the potential for a parallel operation, replicate experimental conditions, and/or integration with other miniaturised components, provide a more reliable diagnostic [19]. Together with the aforementioned advantages, microfluidic immunosensors can achieve the POCT goal identified in the WHO guidelines for biosensors in low-resource settings.

Microfluidics as a field of study of fluidic behaviour and control within structures of micrometre dimensions has been demonstrated to be an efficient platform for integration of several laboratory processes on a single chip [202]. The miniaturisation of the conventional laboratory methods results in lower energy consumption, lower reaction times and better temperature control [202]. Their micro-scale dimensions decrease the required amounts of costly or hazardous reagents (100 nL to 10 μL), while their close architecture reduces the contamination risk. Characterised by a laminar flow and molecule diffusion as mixing method, microfluidics permits optimisation of the chemical reaction rate and enrichment of the products to obtain higher signal-to-noise ratio [203, 204].

Microchannels with complex trajectories can be designed for distinct purposes and can operate in series or in parallel. For example, gradient formation chromatography, electrophoresis systems can be combined to perform multiple tasks and even define sites to deliver stimuli to sub-cellular compartments [203, 204]. The detection methods can vary from optics, electricity, electrochemistry, magnetics [203, 204]. Moreover, these devices can be automated, reducing the operator error risk providing more reliable analysis results [203, 204].

The combination of microfluidic with SERS allows the creation of optofluidic biological assays with an improved performance [7]. The high surface-to-volume ratio of the microchannels leads to an accelerated recognition event between the antibody and antigen which, in turn, allows the development of biosensors with a faster response. Furthermore, the microfluidics network provides a continuously flowing environment, able to generate homogenous mixing conditions, favourable to heat dissipation, and reduces nonspecific adsorption of SERS tags. This allows reduction of the variability inherent in SERS assays, caused by the lack of control over factors such as NP aggregation, size and analyte distributions on the detection surface. Consequently, the SERS-microfluidic devices have increased sensitivity and quantification accuracy [7, 17, 200, 205].

SERS-based microfluidic platforms represent an important advance, since the microfluidic chip fabrication know-how can automate the biological assay process. Steps of adding sample solution, wash buffer and polyclonal antibody-conjugated SERS tag suspension can be automated. Thus, not only can the microfluidic chip replace a tedious human operation and reduce the operator error, but it can also increase the high-throughput biosensing through multiple SERS detection channels [7, 17, 200, 205].

It should be highlighted that the miniaturisation of the optical sensing device is a crucial aspect in medical diagnostics. Thus, although microfluidics provides the ability to develop a small detection platform, most Raman spectrometers are external to the microfluidic device and comprise large optical components (lasers, microscopes, monochromator, and detector). When microfluidic devices and portable Raman spectrometers become integrated into one single system, it will be most likely to become a powerful and robust next-generation biomedical diagnostic tool [7, 17, 200, 205].

### Choice of the material

**Fig. 10:**
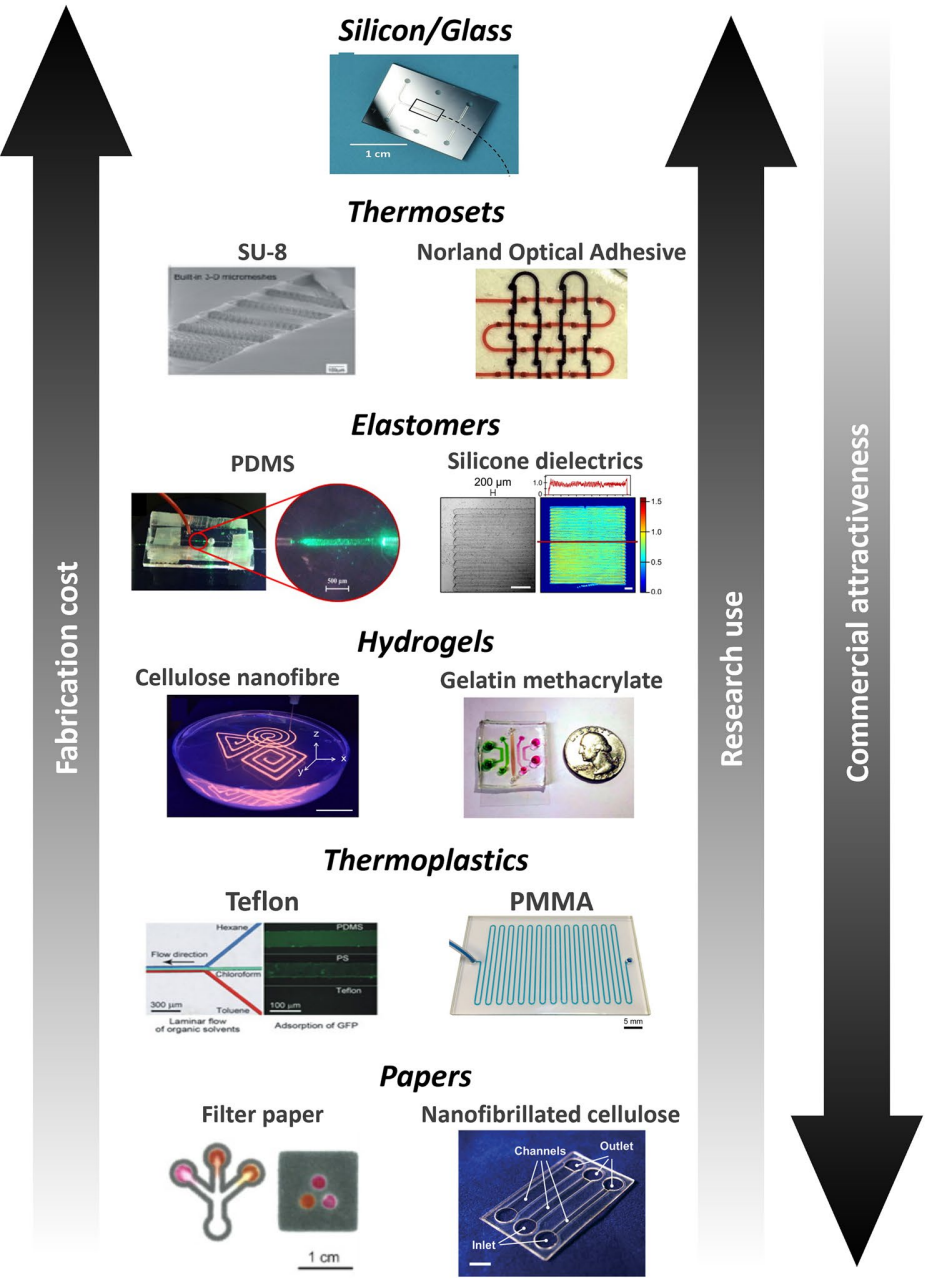
The most promising materials for microfluidic chip fabrication and comparison of the resulting devices’ cost. Reprinted (adapted) with permission from [218] from [214, 219–221]. Copyright 2017 American Chemical Society. Thermosets, inorganic materials and hydrogels have properties suitable for the research level use, while paper may constitute the most spread material in commercial microfluidics. Reprinted (adapted) with permission from [204]. Copyright 2013 American Chemical Society. Reprinted with permission

The function of a microfluidic device is greatly dependent on the material used as the basis of the structure. The choice of the material will depend on the application, as each material has its own advantages and disadvantages (Fig. 10) [203, 204]. Table 4 presents an overview of the main materials for microfluidic chip fabrication, comparing their fabrication processes, stability, price, and main advantages/disadvantages.

**Table 4:** Main materials used for microfluidic chip fabrication

Materials/features	Silicon/glass	Polymers				Paper
		Elastomers	Thermosets	Thermoplastics	Hydrogels	
Examples	–	PDMS	SU-8	PMMA, polystyrene		
Microfabrication technique^a^	Wet and dry etching	Casting	Photolithography	Thermomoulding		Photolitography, printing
Resolution limit	< 100 nm	< 1 μm	< 100 nm	~ 100 nm	~ 10 μm	~ 200 μm
Mechanical stability	Very high	Low	High	Medium	Low	Very low
Chemical stability	Very high	Low	High	Low	Low	Low
Solvent compatibility	Very high	Low	High	Medium to high	Low	Medium
Thermostability	Very high	Medium	High	Medium to high	Low	Medium
Hydrophobicity	Hydrophilic	Hydrophobic	Hydrophobic	Hydrophobic	Hydrophilic	Amphiphilic
Optical transparency	No/High	High	High	Medium to high	Medium to low	low
Oxygen permeability^b^	< 0.01	∼500	0.03–1	0.05–5	> 1	> 1
Scalability	Medium	Low^c^	Low	High	N/A	High
Cost	High	Medium	Medium to high	Medium to low	low	Low
Main advantages	Excellent chemical, mechanical and thermal resistance High thermal-conductivity	*Once the high-resolution mould is made, multiple devices can be fabricated outside the clean room*				Low cost
		Rubber-like elasticity (useful to form pumps and valves Low investment cost	High aspect ratio features High optical transparency	Low cost in mass production		
Main disadvantages	Expensive Laborious and time-consuming fabrication	*Low melting point* *Tendency to adsorb reagents*				Sample evaporation Low sensitivity
		Only suitable for research prototyping	High cost	High investment cost Insufficient gas permeability		
Applicability	Capillary electrophoresis; Analytical and micro-reaction devices	Cell-based studies; Clinical and veterinary diagnostics and POC devices or ‘organs-on-chips’ devices	Platforms requiring high chemical/thermal stability; Moulds for elastomers	Mass-scale production of conventional microfluidic devices		Colorimetric analysis; Mass-scale production

^a^photosensitive glass can be considered as thermoset

^b^Oxygen permeability is beneficial to long-term living cells studies, but the loss of water may lead to a shift in pH and disturb the chemical analysis

^c^Only suitable for research prototyping because is difficult to scale up (cost per unit does not decrease) [204]

Originally, materials such as silicon and glass and technologies frequently used in the semiconductor industry were adapted to fabricate microdevices with excellent mechanical, chemical and thermal stability [204]. However, silicon poses some problems related to not being an electrical insulator (with consequences in electro-osmotic pumping), the tendency of biomolecules to adhere, and the lack of transparency which hinders its application in optical sensing [204]. Glass is transparent, but the bonding procedures need extreme temperatures and voltages [206]. The fabrication cost and taxing processes used with these materials led to adoption of polymers, a broad class of softer materials with a wide range of material properties. Their gas permeability, optical transparency and electrical insulation are important for biological applications specially in cellular assays. The possibility of reducing the cost per unit (in the range of 0.2–2 $cents\cdot {cm}^{-2}$ for polymethylmethacrylate (PMMA) compared to 10–20 of corning glass) and scalability, allied with the various properties depending on the chosen polymer, resulted in polymers becoming the most used class materials for microfluidics, displacing silicon and glass. Thus, polymers became especially important in diagnostic devices. Polydimethylsiloxane (PDMS), a silicone-based elastomer, is by far the most commonly used material in microfluidic immunoassays because it is flexible, optically transparent, biocompatible, and has low-autofluorescence properties [204]. However, PDMS has a hydrophobic nature demanding the modification of its surface from hydrophobic to hydrophilic whilst maintaining its transparency. There several reported options for channel surface treatment to decrease hydrophobicity including plasma treatment [207], polymer grafting [208], biopassivation with lipid bilayer [209] or polymer coating of PDMS with PVA [210]. Nevertheless, the surface chemical instability still hinders the application of PDMS-based microfluidic devices in biological assays [207].

One considerable pitfall is that, due to their chemical composition and structure, polymers can be Raman active materials (*e.g.,* PDMS) [203, 204]. Materials such as calcium fluoride would provide a clean signal due to their IR transparency, but the cost and available microfluidic techniques makes this material difficult to work with [211, 212]. To overcome this challenge, confocal Raman microscopy can be used to focus on the volume inside the microfluidic channel [203, 204].

The use of cellulose-based materials also contributes to the adoption of sustainable concepts, urgently needed in the new worldview of modern society [213]. The interest and increasing trend of using cellulose derives from its large abundancy, renewability, biodegradability, biocompatibility, and hydrophilicity [44, 213]. Among different cellulose materials, nanofibrillated cellulose has been used as substrate material to fabricate electronic devices [44], SERS [214], photoluminescent [44], and cell culture platforms [214]. Notably, a transparent, low-cost, easy to fabricate, and reusable material is highly desirable to better meet the requirements of POCT and possibility to integrate in microfluidics devices. Cellulose-based hydrogels not only represent an attractive choice due to low energy consumption during fabrication and their smooth surfaces, but also as a substrate for immunoassays due to combining cellulose biocompatibility with other fascinating properties such as high-water absorption capacity, stretchability, moldability and stimuli-responsiveness from the hydrogels [215, 216]. Paper has become an increasingly popular material for biosensors, due to its biocompatibility and fabrication cost. Also, paper is especially suited for optical detection methods, since the whiteness of the paper provides the necessary contrast to the colorimetric assays [217]. Paper-based biosensors can be fabricated using several types of papers including cellulose, cation-exchange and anion-exchange paper and bacterial cellulose [217].

### Microfluidic device preparation methods

The first-generation chips were made from silicon and glass in a clean room environment, based on well-established microfabrication techniques that included photolithography and wet or dry-etching methods [23, 204]. The second generation focused on alternative, polymer-based materials. Polymers such as PMMA [222], cyclic olefin copolymer [223], PS [224–226] and PDMS [197, 227] provide a simpler fabrication process (moulding, embossing, and printing) at a lower cost. PDMS-based microfluidics chips are the most common and the fabrication process usually employed is by replica moulding [227, 228]. Using a photoresist (usually SU-8) film spin-coated on a silicon wafer, the photoresist is subsequently patterned by photolithography and then used as a mould for PDMS or other elastomer [227, 228]. This technique provides high fidelity of the intended nano and microstructures and is also a straightforward process suited for small-scale prototyping. Nevertheless, it is time-consuming, generates a significant amount of chemical waste, and is still not able to respond to the great demand for the output of chips in large-scale production. Thermoplastic elastomers, in contrast, can be manipulated by the already full developed industrial polymer manufacturing technologies which simplifies and reduces the cost of mass production [22, 25, 229]. Additionally, PDMS strongly adsorbs biomolecules, later leaching them into the sample flow [230]. These disadvantages hinder the use of PDMS for biomedical applications.

**Fig. 11:**
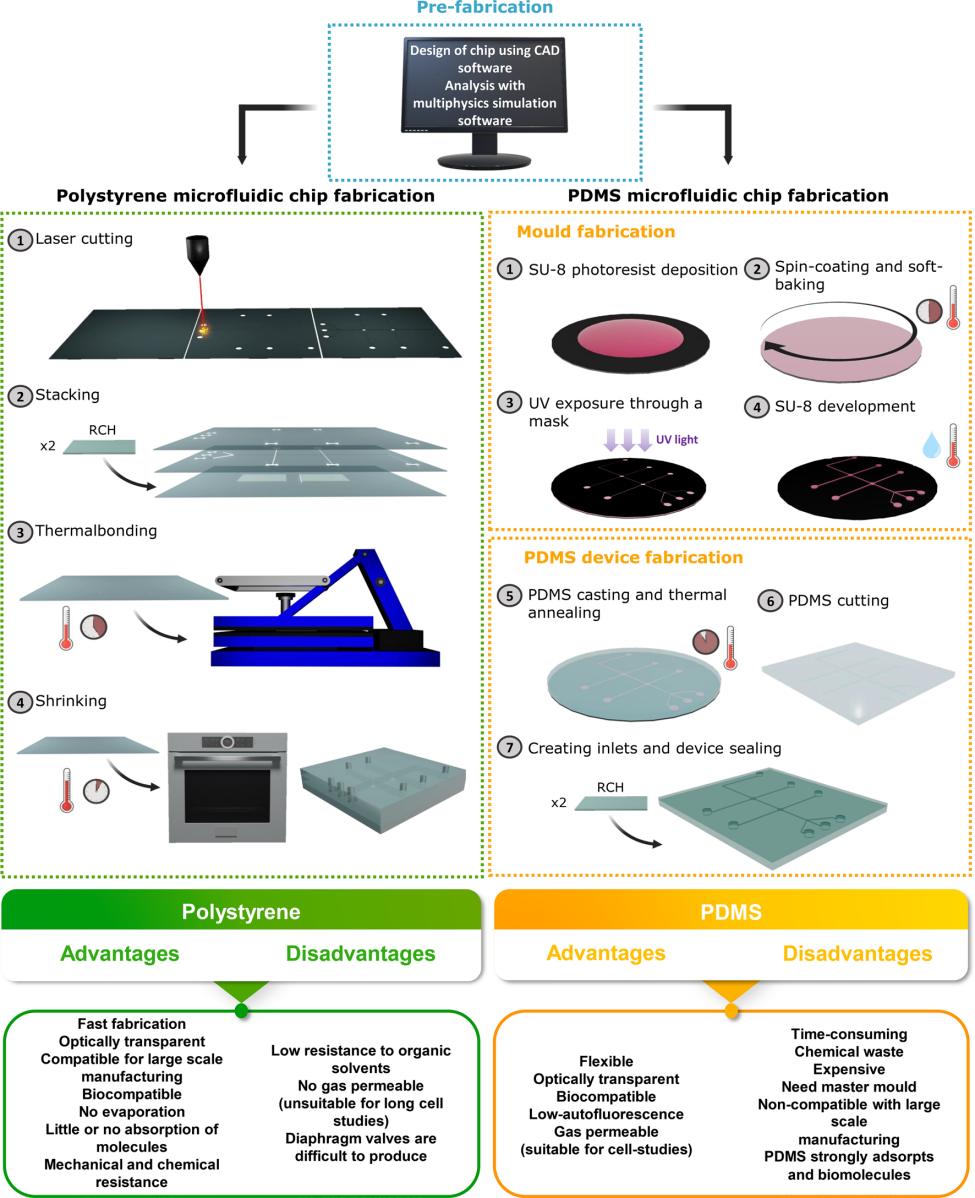
Schematic illustrations of the fabrication process using PS or PDMS as the material of the chip. The pre-fabrication step is common to both fabrication processes. A chip design is drawn in CAD software and performance analyses can be performed in multiphysics simulation software. left: PS microfluidic chip fabrication process. (1) Laser cutting and engraving of PS substrates; (2) Stacking of the layers to form the 3D multilayer device; (3) Thermal treatment with pressure to bond the three layers of the chip; (4) Shrinking the device in an oven. right: (1) SU-8 photoresist application to Si wafer; (2) Spin-coating and soft baking to evaporate the solvent of the SU-8 photoresist; (3) Mask alignment and exposure to UV light; (4) SU-8 development, baking, and rinsing; (5) PDMS casting and thermal annealing; (6) PDMS chip cut and peeling-off; (7) Create inlets and sealing of the chip

Recently, thermoplastics have become an attractive choice to surpass the current limitation of PDMS. Their inherent robustness to mechanical deformation and chemical resistance makes these materials suitable for biochemical microfluidic applications [194, 224, 226, 231, 232]. Microfluidic devices made of these materials can be produced by 3D printing [218, 233, 234], laser ablation [225, 235, 236] or by high-volume fabrication methods such as injection moulding [237], micro-milling [238], and hot embossing [239]. Figure 11 illustrates the comparison between the fabrication procedures of conventional PDMS microfluidic chips and PS-based microfluidic devices using laser ablation.

Laser ablation micromachining has emerged as an alternative method to fabricate microchannels without requiring master moulds [225, 235, 236]. UV lasers were the firsts used, and their laser ablation mechanism resulted in a combination of photochemical and photothermal processes, in which, some chemical bonds of the material are broken directly from photon absorption while others are thermally broken by the released heat from the excited molecules that did not break photochemically [236, 240]. Using $C{O}_{2}$ laser sources, emitting IR radiation at a wavelength of 10.6 $\mu \mathrm{m}$, the ablation occurs solely through photothermal process [222, 236]. Carbon dioxide laser systems have been widely used for rapid and low-cost per unit production of microfluidic systems in a variety of materials including paper [241], polycarbonate [231], PMMA [242], cyclic olefin copolymer [196, 243], polystyrene [222, 225, 226, 235, 239, 242–245], among others. PS, in particular, poses several advantages as it is already the standard material used in cell-culture plates and ELISA applications due to its biocompatibility [246]. PS is optically transparent, inert, rigid, and has a surface that can be easily functionalised [225]. In 2008, Chen et al*.* [244] used pre-stressed transparent PS sheets that retract upon heating. As a result, the pre-etched microfluidic channels shrink isotropically, becoming thinner and deeper (*i.e.* the PS sheet becomes thicker). This process eliminates the need of masks, templates, and the typical microfabrication facilities and consumables that usually result in large amounts of waste. Thus, PS-based microfluidic devices significantly reduce prototyping time and cost. Although promising, PS is still not fully explored in microfluidic biosensors applications [226].

3D printing has blossomed in recent decades, due to the ability to generate a 3D object in a more rapid, affordable, and easier way than other additive manufacturing technologies. Additionally, 3D printers can use several materials with different physical and chemical properties that can be used to create parts that are assembled into a single device [247]. This feature is incredibly valuable for integrating parts such as valves and electrodes through a combination of rigid and flexible materials [247]. This effortless method allows a rapid microfluidic prototyping development at a lower cost than using cleanroom prototyping processes.

3D printing can be accomplished in different ways. Some techniques such as selective laser sintering and fused deposition modelling use melting or softening materials to produce the layers. In the first case, a laser is used to sinter the media and form a solid, while in the latter, a filament is fed through a moving nozzle that deposits the melted polymer onto a support structure. Fused deposition modelling is one of the most used processes, since it is regarded as the less expensive technique to purchase and maintain. Bressan et al*.* [248] for instance, designed a 3D-printed microfluidic device using this technology. Using poly (lactic acid) as the printed part on the top of a PMMA slide, the authors created transparent microfluidic channels to synthesise Ag and Au NPs that were then employed as SERS substrate.

**Fig. 12:**
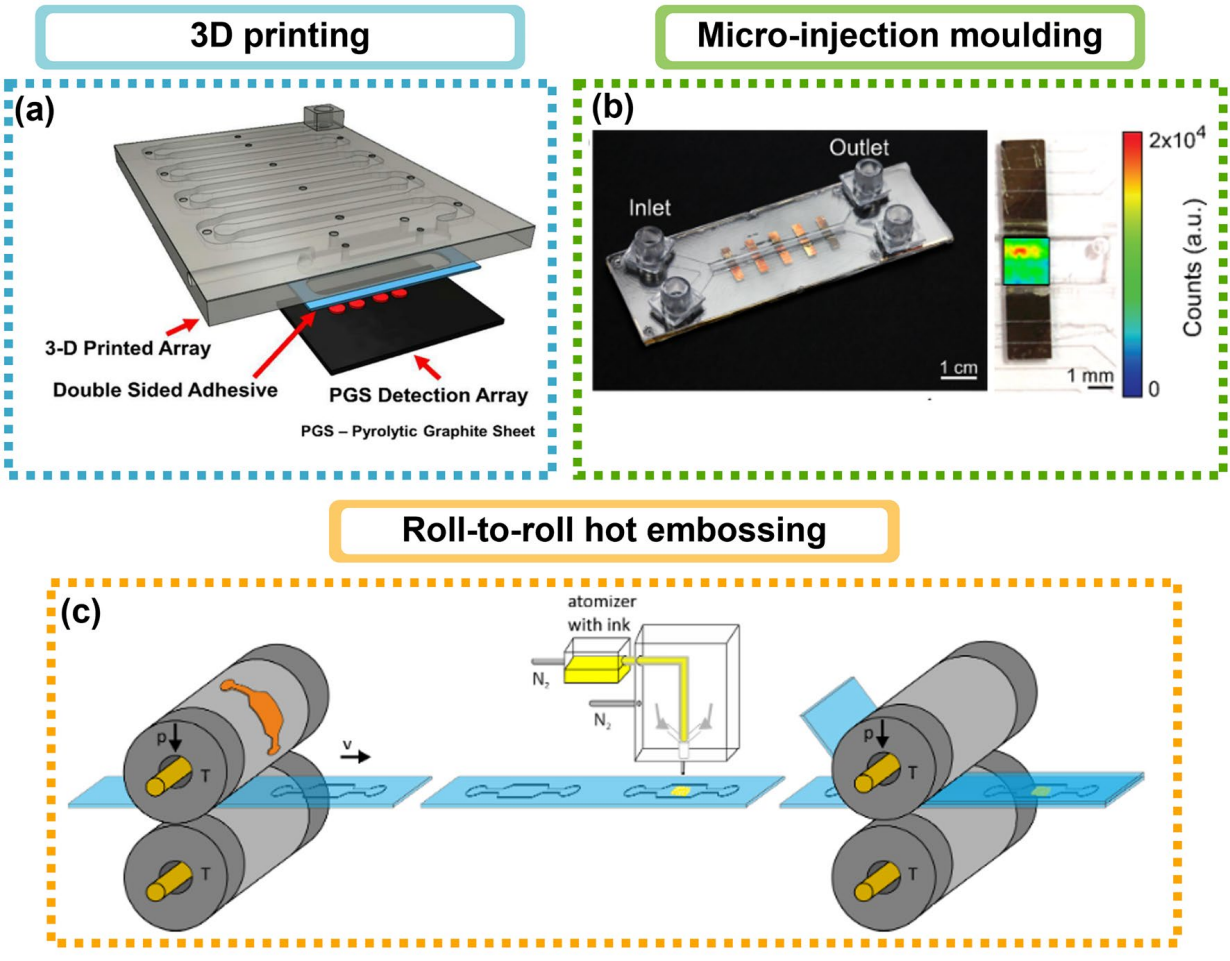
Microfluidic device preparation methods for SERS-based applications. **a** 3D-printed immunoarray with the pyrolytic graphite sheet attached. Adapted from [234]. **b** Left: Photograph of the microfluidic system with the nanostructured bottom layer with Au coating. Right: SERS maps recorded from the sensor array area. Reprinted (adapted) with permission from [253]. Copyright 2018 American Chemical Society. **c** Fabrication process of optofluidic chips for SERS application using roll-to-roll hot embossing of PS foil. Adapted from [249]

Other technologies use liquid materials that are then cured. This is the case of vat polymerisation that comprises stereolithography and digital light processing whereby the liquid photopolymer resin, is turned solid upon UV light exposure. Kadimisetty et al*.* [234] developed a 3D microfluidic electro-chemiluminescent immunoassay printed using stereolithography coupled with a nanostructured pyrolytic graphite sheet (Fig. 12a). This assay not only allowed to reduce the reaction volume for 2 µL *per* sample, but also was able to be completed in 18 min at cost of < 1.00$.

A higher resolution can be obtained with two-photon photopolymerisation, whereby the gel is cured only in places where the laser was focused. In fact, the main challenge in 3D printing for microfluidic applications is to achieve the resolution required for microfluidic channels usually below 100 µm. Moreover, it is difficult to remove the resin/support materials form the microstructures which causes defects that have a high influence on the performance of the microfluidic device. Nevertheless, 3D printing is still in an early-stage development and the potential visioned for novel microfluidic and sensing systems will surely encourage to develop new efficient technologies that tackle the existing challenges.

All the aforementioned methods have however, one main drawback, they are not suited for high volume production. As an alternative, thermoplastic polymer microfluidic devices have been fabricated through methods such as hot embossing and micro-injection moulding. Hot embossing is characterised by a transfer of features from a mould (usually negative) to a polymer substrate by elevated temperatures and pressures. Micro-injection moulding on the other hand, uses a higher pressure to perform the process at room temperature. Consequently, the fabrication time is shortened. When combined with roll-to-roll process, hot embossing and injection moulding can achieve high production volumes at low cost with high reproducibility. One striking example, designed by Habermehl et al*.* [249], how the production of a SERS-based microfluidic device, using aerosol jet printing for the fabrication of the SERS substrate, combined with roll-to-roll hot embossing for the microchannels production in polystyrene foil (Fig. 12c). Viehrig et al*.* [250] on the other hand, used injection moulding and roll-to-roll process to fabricate an ultrathin microfluidic chip with a nanostructured Ag film using a cyclic olein copolymer and a polyethylene terephthalate foil (Fig. 12b). This ultrathin microfluidic device enabled SERS measurements to be performed in much proximity reducing interferences and fluorescence signals. Notwithstanding, these techniques must still perform under vacuum environment and the moulds used are typically fabricated using microfabrication. However, recently, low-cost metal moulds have been exploited using computer numerical control milling and 3D printing [251, 252].

Hence, the techniques available to design a microfluidic device suited for SERS applications are numerous, and the choice between them will depend on the minimum feature size needed, surface roughness, aspect ratio, interference with the sensing system, among others. All these parameters need to be carefully thought and as they will influence the fluid behaviour which ultimately can compromise the assay. Although significant efforts have been dedicated to optimising an approach for a particular application, the production volume, cost, and reproducibility need to be considered for commercial purposes in early stages [229]. This is especially the case for low-resource settings that have shortage of sensitive and cost-efficient systems.

## Challenges and perspectives

The development of SERS tags with applications in bioanalysis has contributed significantly to understanding the physicochemical properties of the NP-biomolecule system that determine the outcome of these tags. Nevertheless, challenges remain unsolved for optimal adaptation to microfluidics-based detection systems, such as obtaining reproducible signals, quantification and calibration of SERS-tags and expansion of multiplex labelling ability.

In parallel, the microfluidics field has seen as enormous growth over the years, with a broad range of applications. Notwithstanding this, microfluidic platforms can be greatly improved by increasing the amounts of laboratory protocols that can be included in one single device. Besides automation, the lifespan of the chip (*e.g.,* possible channel blocking and clogging), the low throughput (*e.g.,* single channel designs), the complex fabrication and limitations linked with material chosen, and the Raman signal of the material’s device are among the challenges to be addressed.

### Reproducible signal of SERS tags

Reproducibility has been a feature much criticised in SERS field. In the case of SERS tags, two factors must be considered: the hotspots and the RR molecules. Uniformity and distribution of both factors can have a tremendous impact in clinical diagnosis and biochemical detection and is one of the reasons why SERS has not yet made the translation into standard detection methods specially in clinical fields.

**Fig. 13:**
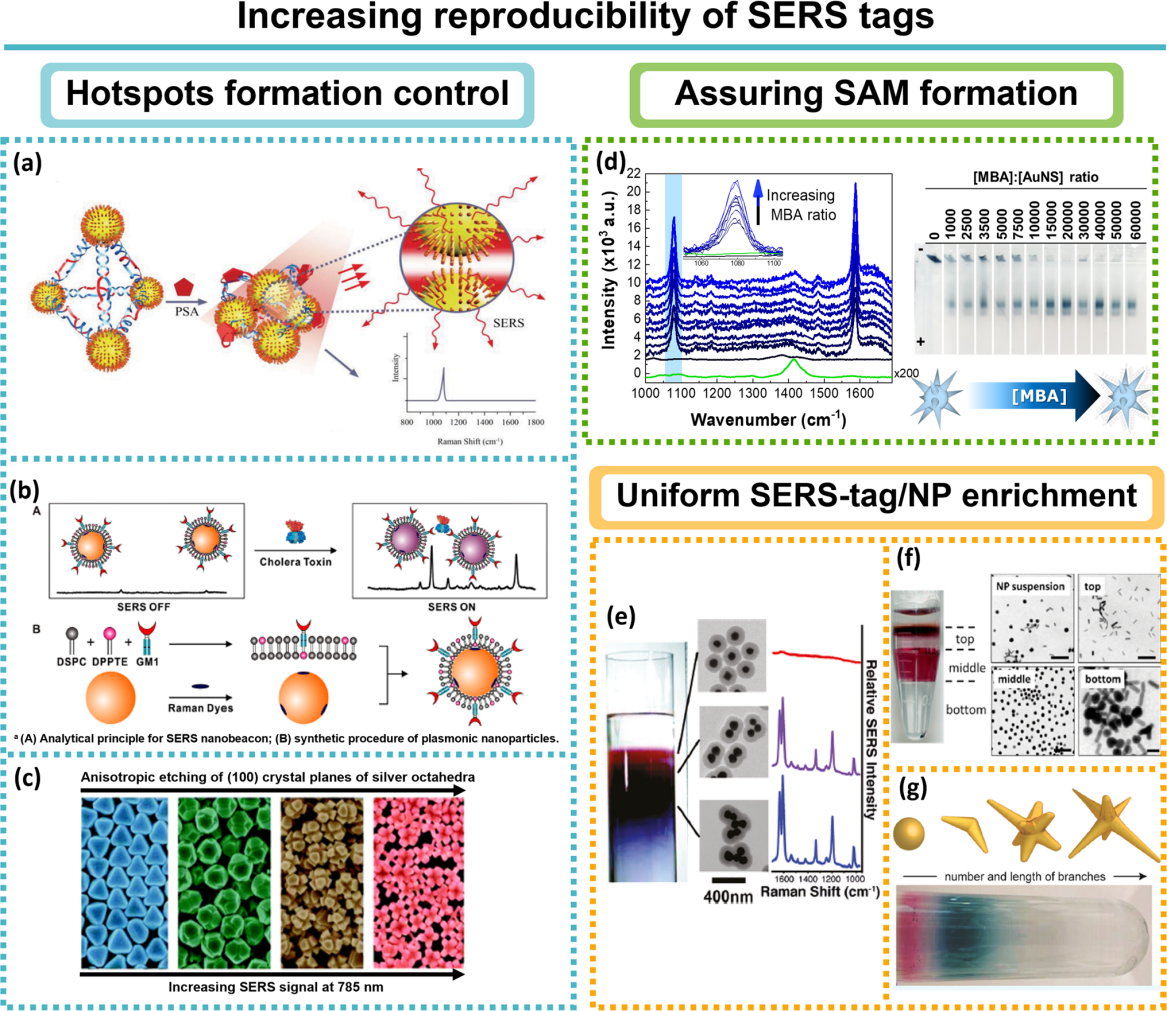
Examples to overcome irreproducibility of SERS tags. Hotspots formation control: **a** SERS encoded Ag pyramids for detection of prostate specific antigen (PSA) disease biomarkers using aptamers. Adapted from [268]. **b** Scheme of plasmon coupling enhanced Raman scattering nanobeacon for cholera toxin detection. Reprinted (adapted) with permission from [167]. Copyright 2016 American Chemical Society. **c** False-colour SEM images following the etching progress of the silver octahedral-shaped nanoparticles. Reprinted (adapted) with permission from [114]. Copyright 2010 American Chemical Society. Assuring SAM formation: **d** Left: SERS spectra of functionalised AuNSs, with increasingly more intense lines at 1079 cm^−1^ (inset) and 1587 cm^−1^, which confirmed changing the capping agent by disappearing citrate lines and appearing MBA lines, as the molar ratio of MBA increases. Right: Photograph of an agarose gel with AuNSs samples functionalised with increasing molar ratios of MBA. Adapted from [260]. Uniform SERS tag or NP enrichment: **e** Photograph of a centrifuge tube after centrifugation of the sample in a surfactant-free aqueous iodixanol linear density gradient. TEM images of the selected fractions whose positions in the centrifugation tube are spatially indicated and corresponding SERS spectra of three selected fractions. Reprinted (adapted) with permission from [267]. Copyright 2011 American Chemical Society. **f** Separation of NPs into an aqueous three-phase system composed of Brij 35, PEOZ, and Ficoll. Small nanorods penetrated slightly into the top phase, small nanospheres migrated to the middle phase, and large particles of both shapes sedimented to the bottom. For each phase, the corresponding TEM images are shown. Reprinted (adapted) with permission from [265]. Copyright 2012 American Chemical Society. **g** Separation of stabilised morpholine-based Good’s buffer, AuNSs by density gradient centrifugation. Adapted from [264]

The precise control over hotspots plays an important role in designing SERS substrates since Fang and co-workers reported that the: “hottest sites (h > 10^9^) account for just 63 in 1,000,000 of the total but contribute 24% to the overall SERS intensity” [254]. Random aggregation of metal NPs was one of the first approaches to increase Raman signal enhancement. However, irreproducible results together with lack of correlation of SERS signal with the exact nanostructure from which it originates, led to abandon this approach. As a result, new controlled aggregation approaches were developed using bifunctional linkers (*e.g.,* polymers [122], oligonucleotides [255], or even the analyte [256]) able to connecting NPs together. These bifunctional linkers are placed in the hotspot, and some can also act as a RR. These approaches mean that, to profit from these hotspots, the molecules must be RRs or competing with the linking molecules for the vicinity of the hotspot. The molecular binding events triggered by small molecules can also be explored to act simultaneously as an aggregation and a detection binding event (see Fig. 13a). As shown in Fig. 13b, cholera toxin, for instance, binds to monosialoganglioside, and can be integrated in RR labelled AuNPs which assemble into cross-linked aggregates in response to cholera toxin [167]. Nevertheless, only small molecules can be used as analytes, so this approach lacks universality.

Controlling hotspot formation by chemical processes increases the possibility for a RR molecule be in vicinity of these strong electromagnetic fields and subsequently a more reproducible SERS signal than simple aggregation of spheres [110]. Two main approaches can generate particles with several hotspots: anisotropic etching [114] and anisotropic growth [113]. Anisotropic nanocrystal growth methods, as mentioned in Sect. 3.1, can provide several hotspots *per* particle. Nevertheless, not all geometries are possible and thus anisotropic etching processes are used to prepare new NP shapes with high yields and purity [114]. Tuning the etchant strength and reaction conditions, Mulvihill et al*.* [114] showed that intraparticle gaps could be created, yielding enhancements of 10^4^–10^5^ (see Fig. 13c). However, these methods still present a higher degree of complexity compared to anisotropic growth and are usually achieved in the presence of a polymer [122] which might impair RR adsorption to the NP when preparing SERS tags.

A reproducible signal is also dependent on the population of the produced SERS tags. Strategies to ensure that all SERS tags yield the same SERS enhancement include SAM-coating [128] or post-synthesis purification of bioconjugates [257–259]. The concentration of RR is frequently confirmed by reaching an equilibrium concentration between colloidal stability and by noting that the SERS spectrum intensity is unchanged [58]. However, the work of Oliveira et al*.* [260] highlighted that, despite using a concentration able to exhibit a high SERS intensity and a red-shift of LSPR, the formation of a RR monolayer should be assessed by complementary techniques (see Fig. 13d). The authors used agarose gel electrophoresis to verify the correct amount of the negatively charged MBA functionalisation agent necessary to fully cover the NPs [260]. Other authors also demonstrated the advantages of using gel electrophoresis to separate DNA/polymers functionalised NPs by size, shape and charge [257, 259]. Most of the techniques, however, such as size-exclusion chromatography [261], diafiltration [262], centrifugation (in particular density gradient centrifugation) [261, 263–265], have been used to separate metal NPs according to size and shape due to sedimentation-coefficient differences. Chen and co-workers [266], for instance, used differential centrifugation with a high-density CsCl solution to distinguish SERS tags formed with nanoclusters of different sizes assuring a more consistent SERS enhancement. Others used a high-viscosity density gradient medium, to obtain an enriched solution of SERS tags in one aggregation state and a diminished monomer population (see Fig. 13e) [267]. Separating NPs of completely different shapes such as spheres from rods with 99% of yield is somehow easy to achieve by performing a centrifugation, whereas separating nanostar suspensions, an intrinsically heterogenous sample, can be challenging [263]. Interestingly, Chandra et al*.* [264] used a linear sucrose gradient and was able to sort nanostars based on branch length and number (see Fig. 13g). Thus, these colloidal spatial isolation techniques can be extremely helpful to reduce the ambiguities in calculating and interpreting the respective SERS EFs.

Improving the reproducibility of the bioconjugation process has been explored by employing methods based on click chemistry to assure that all the specific-target ligands exhibit the same orientation and consequently, the same specificity towards the analyte [140].

### SERS-tags quantification: calibration of SERS intensities and enhancements

The signal observed from an analyte in Raman measurements is proportional to the concentration in the probed volume, and thus it is possible to directly correlate the intensity of a vibrational band as a function of the analyte’s concentration. SERS signal response from SERS tags bound to analytes also follows the same behaviour [7, 8, 14, 52, 76, 81, 269]. Despite being the most common method used for quantification, it is difficult to reproduce the absolute intensity of signals even from the same sample. Tian et al*.* [270] showed how the amount of measurement points can influence the relative standard deviations and congruently limits of detection and quantification. The SERS mapping quantification strategy led to an improvement of the SERS signal stability because it considers the sample’s distribution inhomogeneity reducing the relative standard deviations by 50%. Whilst this approach minimises the problem of quantification, the time required for analysis and strategy makes it not sufficient or applicable to all SERS systems. Therefore, improvements in SERS quantification are almost always followed by selecting a suitable calibration method for real sample analysis [271]. Calibration of Raman signals in conventional Raman analysis is a typical procedure to minimise the variance of Raman instrumental and experimental factors, including variation in the optical alignment, changes in excitation laser power or detector, and variation in the positioning of the sample [271]. Adding a standard-based calibration method corrects the effects of changes in scattering from solutions due to turbidity, or inner filter effects from absorbing compounds and differences in focus due to non-uniform sample morphology from solids. Calibration methods can be external or internal [269]. External calibration provides high-throughput analysis with quantitative results in a short time, but is usually performed in different matrices of the testing solutions from real samples, which can decrease the calibration results accuracy [272]. Addition of internal standards into sample solutions can partly neutralise the influence of the sample matrix [273], and provide SERS signals that can be correlated with a tag signal reducing the fluctuations of near‐field enhancement among different hotspots and variations of instrument factors [274]– [276]. Therefore, internal standards should be sought in future works.

Internal standards can be categorised in three groups: (1) non-SERS-active compounds with unenhanced signals strong enough to be detected in the presence of the surface-enhanced analyte’s signal [273]; (2) compounds that can generate enhanced signals similar to those of the analyte, with one or more bands in spectrally silent regions [274, 276]; and (3) chemicals with structures similar to those of the analyte or an isotopically substituted form of the analyte itself [139].

**Fig. 14:**
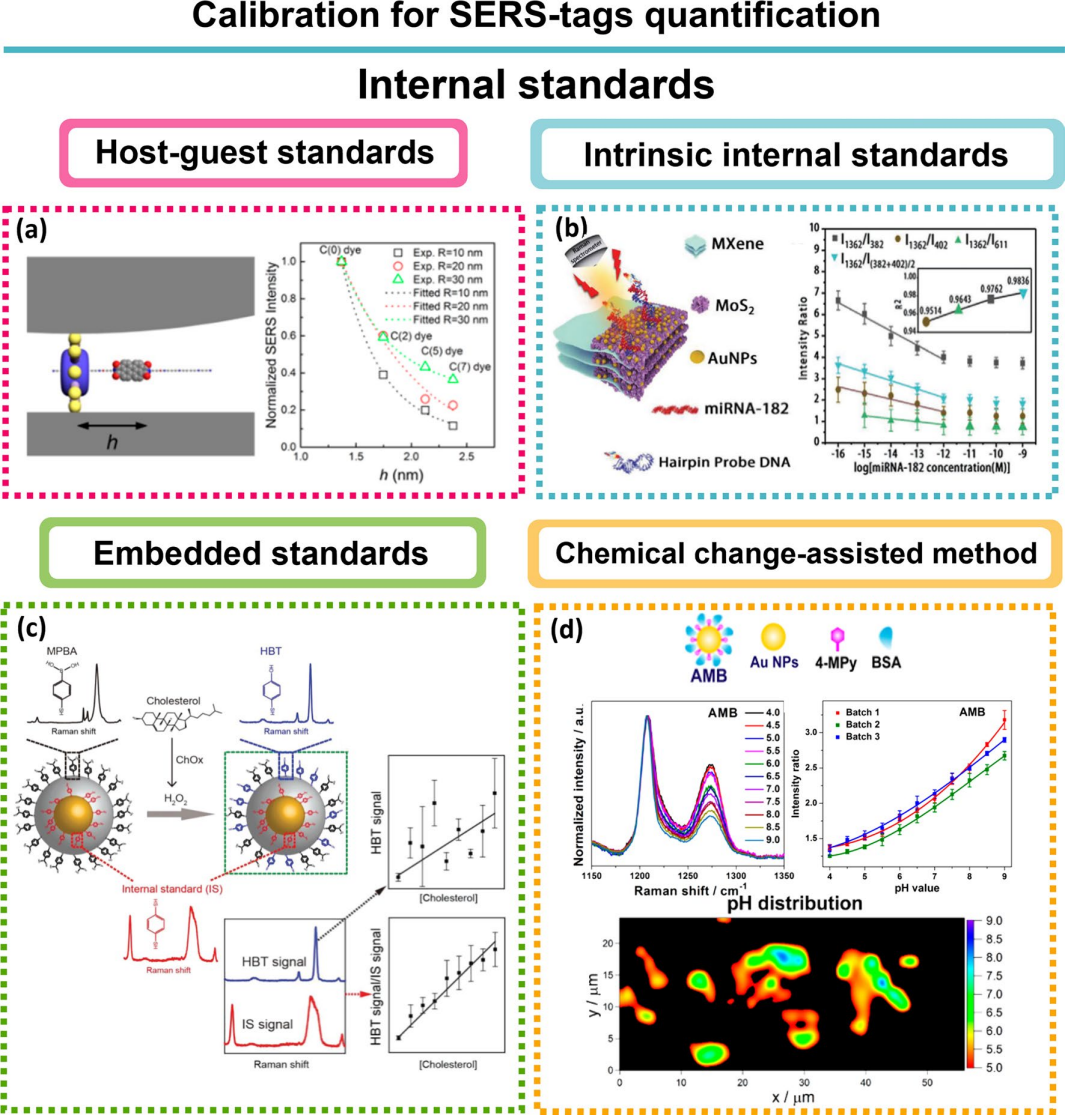
Calibration for SERS-tags quantification by internal standards. Host–guest standards. **a** Positioning of the spermine-C(n)-perylene bisimide moiety with respect to the centre of the hotspot. Normalised SERS intensities of the 1300 cm^−1^ band as a function of the distance, h, n = 10. Adapted from [279]. Intrinsic internal standards. **b** Left: synergistic calibrated SERS strategy based on MXene/MoS_2_@AuNPs for the ultrasensitive detection of cancer-related miRNA-182. Right: SERS intensity ratio and fit curves of ratiometric value generated from MXene/MoS_2_@AuNPs with miRNA-182 at different concentrations. Adapted from [282]. Embedded standards. **c** Quantitative detection of cholesterol via the Raman spectral fitting method using SERS tags with the embedded internal standard. Adapted from [283]. Chemical change-assisted method. **d** top: SERS spectra of Au-(4-MPy)-BSA pH nanosensor in PBS solutions of various pH values. Au-(4-MPy)-BSA pH nanosensor of different batches shown with the intensity ratio from 1208 to 1274 cm^−1^ as a function of pH values. Bottom: pH image of a CaSki cell after incubation with Au-(4-MPy)-BSA pH nanosensor for 4 h. Reprinted (adapted) with permission from [287]. Copyright 2014 American Chemical Society

Ideally, these internal standards should have similar chemical properties to the targets so that they respond to the perturbations of the detection environment. Conversely, searching for internal standards with similar chemical properties can trigger a competition for adsorption between targets and ISs for the hotspots, possibly causing false positive results and consequently, hindering the widescale applications of this method [76]. Using molecules simultaneously as internal standards and host takes advantage of this competitive adsorption process. The molecule in the hotspot can selectively capture the analyte in a stoichiometric relationship through host − guest interactions and thus can be correlated with analyte concentration. In host–guest interactions, both the analyte and the host molecule experience the same EM field, making a IS sensitive to the environment. Cucurbit [n]uril is an example of a macrocyclic host molecule, consisting of *n* glycoluril repeat units, that can act as a selector of molecules with no affinity to the NP surface, or not soluble in water, and as a RR (see Fig. 14a) [277]. Due to its sub-nanometre dimensions, it can be placed within a hotspot and attract the analyte with a strong resultant SERS signal. It has been used to selectively capture RRs such as rhodamine 6G [277, 278], and spermine-C(n)-perylene bisimide [279], but also relevant biomarkers such as neurotransmitters (dopamine, epinephrine and serotonin) [280] and uric acid [281].

Instead of anchoring molecules directly on the metal surface that might be easily detached or occupying the detection site by competition events, Liu and co-workers [282] developed a synergistic calibrated SERS strategy based on ternary system composed by MXene/MoS_2_@AuNPs (see Fig. 14b). In this way, the authors created an ultrasensitive detection system without using extra internal standard molecules [282].

Another way to include an internal standard without inducing a competition for the target analyte is to embed the standard molecule in the core–shell NPs. For example, Jiang et al*.* [283] developed Au–Ag core–shell NPs with embedded 1,4-BDT (Au@BDT@Ag) as the SERS substrate and functionalised with 4-mercaptophenylboronic acid (4-MPBA) and conjugated with cholesterol oxidase (ChOx) (see Fig. 14c). 4-MPBA selectively and efficiently reacts with hydrogen peroxide converting it into 4-hydroxybenzenethiol (4-HBT) and ChOx quantify cholesterol via H_2_O_2_. Thus, the internal standard-based calibration method can quantitatively detect hydrogen peroxide and cholesterol. The embedded IS method is perhaps the most prevalent in literature due to effectively correct the signal fluctuation and has been applied over a variety of different morphologies [283–285]. However, colloidal stability has been one of the drawbacks identified for embedded standards in core–shell substrates [283–285].

SERS tags that have an intrinsic internal standard represent a simpler approach than embedded internal standards [89]. This is because, in intrinsic IS methods, the SERS signal originates from the raw materials during the preparation of SERS tags, and not from the probe molecules deliberately added for embedded IS methods.

Alternatively, it is possible to use molecules which present vibrational bands sensitive to the ion coordination event, altering their associated Raman bands intensities, whereas the other bands of insensitive bonds remain nearly unchanged regardless of the variation in field enhancement caused by local differences in the substrate. Consequently, these unchanged bands allow to quantify the analyte by accessing to their height or band area ratios. Monitoring local intracellular pH using pH-responsive probes such as MBA [89], p-aminobenzenethiol [286] and MPY [287] is one of the applications that takes advantage of this chemical change-assisted method (Fig. 14d). Other examples include trimercaptotriazine-modified AuNPs or terpyridine-modified AgNPs that not only exhibit strong SERS signals but are also sensitive to the presence of heavy metal ions. Zamarion et al*.* [288] used these NPs to selectively probe Hg^2+^ and Cd^2+^ ions and Tsoutsi et al*.* [289] detected copper and cobalt at ultra-trace levels simultaneously. In both cases, the authors used bands ratios avoiding the need of using extra internal standards.

Although a universal standard detection method has not been adopted, the usage of references always leads to an improvement in the linear fitting degree which indicates that the accuracy of quantitative SERS measurements has been significantly increased [274–276].

## Improving multiplexing capability

The requirement for developing multiplex assays is related to the fact that many diseases cannot be diagnosed from a single biomarker [5]. Cancer, for instance, is a diverse disease and the correct diagnosis depends on multiple biomarkers being simultaneously detected [290].

The ability of a mixed suspension of SERS tags to be used for simultaneous detection of multiple targets with high sensitivity and selectivity is the main motivation for the development of encoded SERS tags. Although several papers mention two or three reporters with non-overlapping characteristic bands, the complexity that comes from using more reporters, can impair the peak integral method to validate the SERS tags identification [7, 15, 62]. To date, few papers have reported a library and systematic analysis of several SERS tags encoded with different reporters. In part, this is due to the different chemical nature of the involved RRs (*e.g.* aromatic thiols *versus* chromophores dyes) making difficult to design a collection of SERS tags feasible and scalable over a wide number of RRs.

**Fig. 15:**
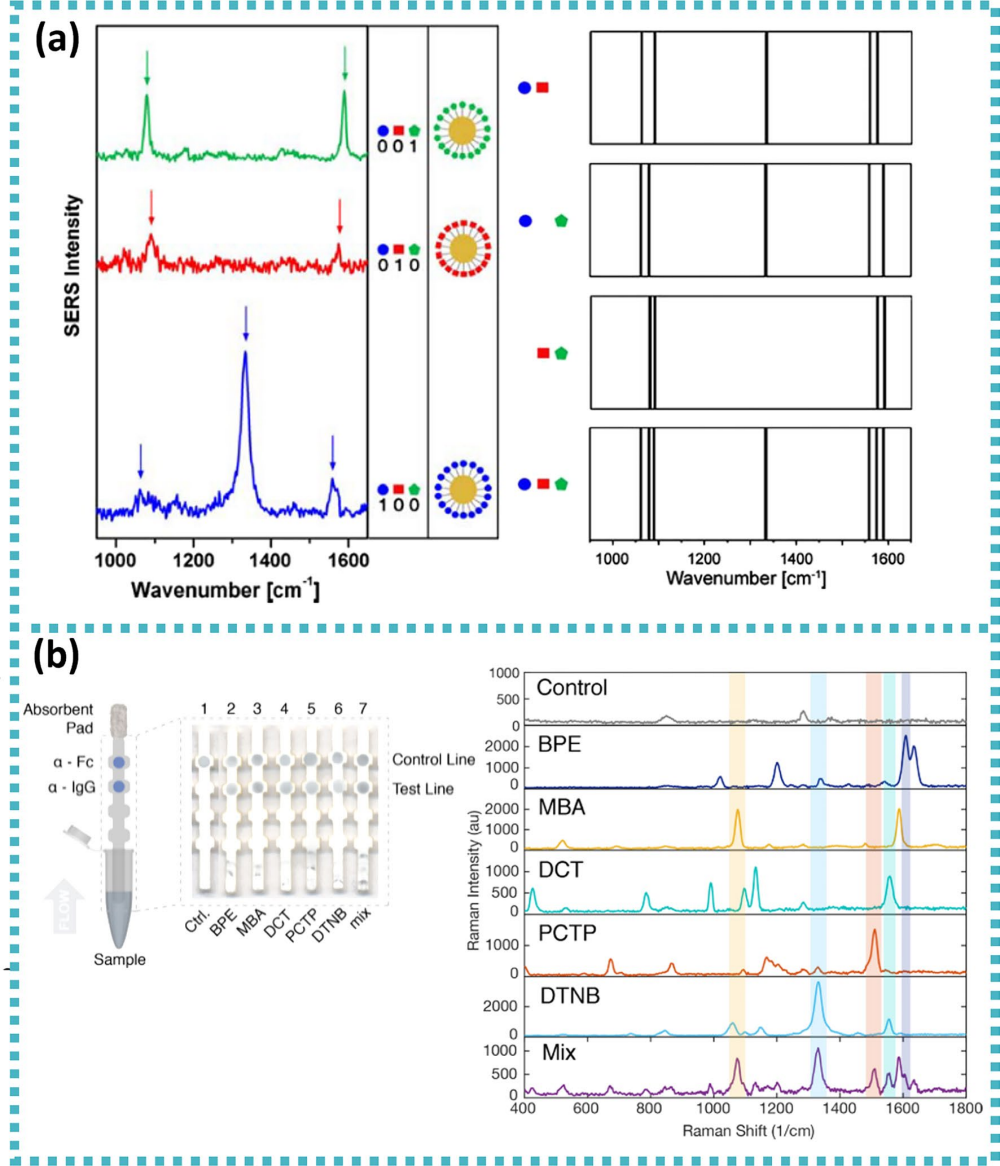
Examples of multiplex capability from SERS-based assays. **a** Left: SERS spectra of one-component SAMs on AuNP: 5,5′-dithiobis(2- nitrobenzoic acid) (DTNB) (blue), 2-bromo-4-mercaptobenzoic acid (BMBA) (red) and MBA (green). Right: Bar code diagrams for SERS spectra of one-, two-, and three- component SAMs on AuNPs. Adapted from [293]. **b** Left: Dipstick flow immunoassay scheme and the resulting strips from running individual SERS tags with the five reporters (strips 2 − 6) and strip from the mixture of all nanotags (strip 7). Strip 1 is the negative control. Right: SERS spectra of the negative control, the five SERS tags, and the mixture (mix). Adapted from [294]

Gellner et al*.* [128] increased the multiplexing capacity by inserting an additional parameter to the number of RRs—the stoichiometric ratio (see Fig. 15a). Mixing RRs to form a multicomponent SAM in different ratios, yields a bar code of only three reporters that could generate seven different signals [128]. However, these kinds of methods can only be applied if the RRs used have equivalent anchor groups. To achieve multiple active SERS tags, the design strategy must be independent of the chemical nature and size of the RR used. Few examples have been reported [137, 151], Mir-Simon et al*.* [151], for instance, designed a synthetic strategy capable of testing 31 different codes. Nevertheless, using an increasing number of reporters will inevitably lead to overlapping bands making difficult to identify accurately individual tags (see Fig. 15b). Multivariate curve resolution methods and machine learning can aid to overcome these pitfalls, as well as the fluorescence interference, as will be described in Sect. 6.4.

Instead of using different codes, it is possible to divide them spatially. Fabrication of arrays provide the ability of using few SERS tags but spatially addressable, thus enhancing the capability of multiplex [291].

Combining sensing tags is another option to increase the multiplex ability. Dual modal nanoprobes resulting from a mixing two different RRs with two fluorophores allows four codes suitable for cell imaging (see Fig. 15c) [292]. If, instead of fluorophores, QDs are used, a larger encoded capacity is obtained [13]. QDs have narrower emission bands and thus produce more colours. When combined with SERS tags, the number of available codes theoretically increases to ${2}^{m+n}-1$, being $m$ the number of QDs and $n$ the number of SERS tags [13]. The multiplexing capacity of different reporters is shown in Table 5.

**Table 5:** Type of labels with physical processes specified for each type and the multiplexing capacity offered by these approaches [69, 82]

Detection principle	Labels	Full width at half-maximum (nm)	Multiplex capacity
Electronic absorption	Dyes	> 50	~ 1–3
Fluorescence emission	Molecular fluorophores	> 50	~ 1–3
	Quantum dots	~ 20	~ 3–10
Raman scattering	SERS reporters	0.5–2	~ 10–100

### Multivariate curve resolution methods

Univariate quantification is based on calculating the area of a well-defined SERS vibrational band and correlate it to the concentration of the target analyte. Whilst caution is needed to apply the baseline and to subtract the background, this method usually works well when analyte’s SERS signal is clean *i.e.,* unique, and not crowded or overlapped with other spectral features. The situation changes when the analyte is within a complex matrix or when the goal is to perform quantitative multiplexed analysis. Multivariate analysis (MVA) techniques use the whole spectrum, or a subset of the spectral features as input and simplifies the multivariate data into a result with lower dimensions and readily interpretable. These MVA methods are included in the machine learning (ML) group and their role in spectral analysis has seen a prevalent increase in literature. MVA have been used in many analytical chemistry fields including Raman spectroscopy for many years [295, 296] whereas their application within SERS, especially in SERS-tags, for quantitative analysis is comparatively less well explored [297]. A recent review provides an overview of the most common machine learning methods for treating Raman and SERS spectral information and their applications [297]. Thus, in this section emphasis will be made towards examples applied for SERS tags.

**Fig. 16:**
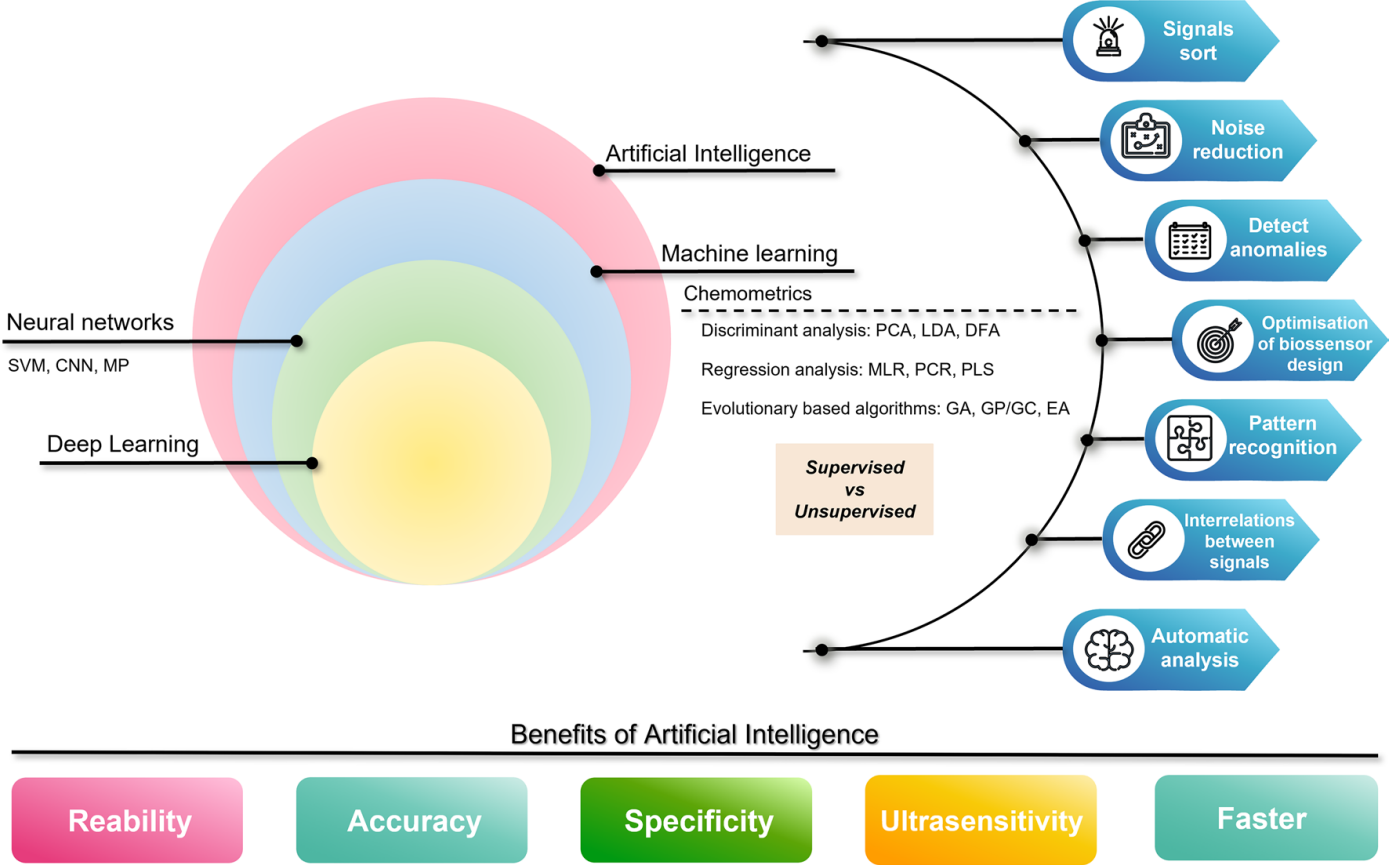
Relationship between artificial intelligence, machine learning, and deep learning, a few of the most used algorithms in the literature are named and the function these methods can accomplish (blue boxes). Chemometrics: discriminant analysis: *PCA* (principal component analysis); *LDA* (linear discriminant analysis); *DFA* (discriminant function analysis); regression analysis: *MLR*: multiple linear regression; *PCR* (principal component regression); *PLS* (partial least-squares); evolutionary based algorithms: *GA* (genetic algorithm); *GP/GC* (genetic programming and computing); *EA* (evolutionary algorithm). Neural networks: *SVM* (support vector machine); *CNN* (convolutional neural network); *MP* (multilayer perceptron). Benefits of artificial intelligence techniques brought to biosensors field

An important note to bear in mind while reading this section is that there is an overwhelming number of machine learning methods and their classification changes from report to report. A general scheme of the artificial intelligence universe is represented in Fig. 16.

MVA may be exploratory analyses or multivariate regression based. Exploratory data analysis allows to extract a relationship between groups of samples with no prior information which means that they are unsupervised [298]. Principle component analysis (PCA) is a very common chemometrics approach that is used to explain the variance in data [299, 300]. Ever since Le Ru et al*.* [301] used PCA to demonstrate single-molecule detection, PCA and other exploratory analyses have been demonstrated for identification and discrimination of signals from cancer cells [302, 303], DNA classification [304], bacteria [305], multiplex SERS tags mixtures [130] and even analyse biochemical components and produce a cell culture environment 3D image [306]. Other works might profit from using more than one MVA method. For instance, PCA in combination with discriminant analysis (DA) as well as ratio analysis, showed the ability of discriminate between cancer and healthy cells as well as biomolecular detection up to a single-cell-level detection using a ZnO-based SERS assay [72]. The advantage of using these methods is their ability to organise the data based on score values that can be used to construct an image in which each identified biochemical contribution is described by the loading plot. Additionally, the loading plots can exhibit a quantification trend and even identify outliers that might arise from sample contamination or photodecomposition products or other analytical artifacts. However, in some instances, the interpretation may be difficult since the identified components in the spectrum do not necessarily represent a single probe [307].

More powerful methods of analysis belong to *supervised* learning methods. The goal of these methods is to ascertain whether measured SERS spectra can be correlated with the targets and once calibrated, is possible to predict the quantity of unseen samples accordingly with the targets. Partial least squares regression (PLSR) has been one of most applied chemometrics supervised methods to quantify a wide range of analytes [296, 308, 309]. Lim et al*.* for instance, applied a PLS model in an immunosensing system to quantify three unknown biomarkers rapidly and accurately from the Raman spectra instead of the conventional calibration method [310].

**Fig. 17:**
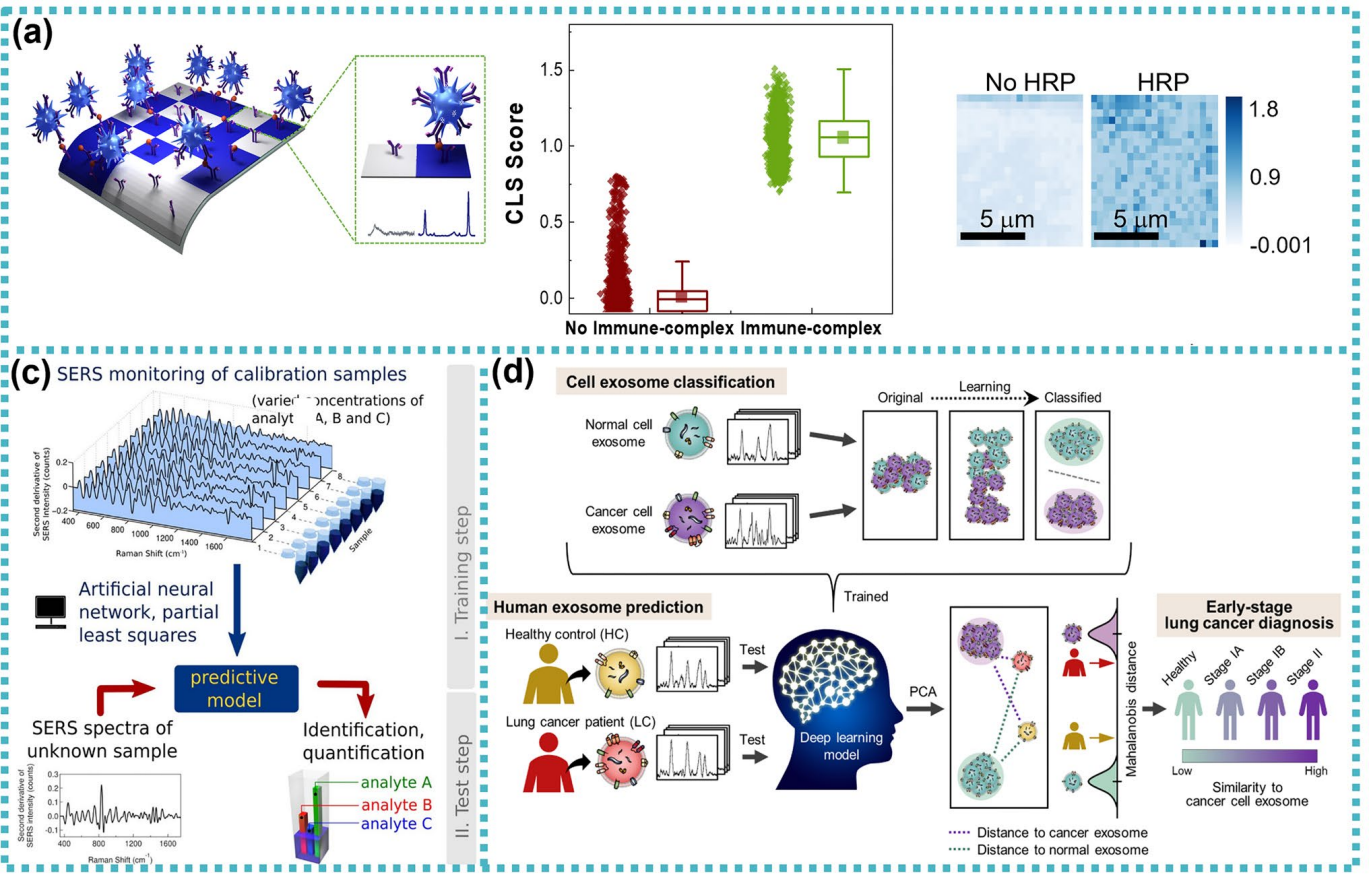
Multivariate analysis methods. **a** Selectivity response of the immunoassay based on CLS results to quantify the contributions of a negative response obtained when using an irrelevant antigen and positive detection when HRP was used. SERS maps for the HRP sensitivity study: control sample where no HRP was added to study the non-specific binding between the capture antibody and the SERS-tags; a sample with the HRP added and consequently bound and identified by SERS-tags. Adapted from [322]. **b** SERS data collected from samples to calibration and used to “train” the data mining methods and build a predictive model. In the final phase, SERS spectra of unknown sample is then collected and analysed using the predictive model to obtain a result. Adapted from [280]. **c** Illustration of deep learning-based circulating exosome analysis through classification for lung cancer diagnosis using exosomal SERS signal patterns. Reprinted (adapted) with permission from [319]. Copyright 2020 American Chemical Society

The ability of performing multiplex SERS assays has been a constant motivation in SERS bioanalysis [7]. Nevertheless, SERS tags signals still need to prevail over strong background Raman signals from cells containers, tissue autofluorescence, or other sources [307, 311]. Moreover, although RRs have narrow linewidths, the higher the number of SERS tags, the higher the probability of overlapping. Multivariate curve resolution (MCR) methods permit to resolve mixtures to identify individual meaningful components within a sample and quantify each component. These data mining techniques have been identified as a crucial step in achieving reliable data. For instance, Tan et al*.* [312] were able to mitigate the influence of high background Raman or fluorescent signals in biological detection using two MCR methods, including negative matrix factorisation and classical least squares. They were able to resolve four different SERS tags (each with a different Raman reporter) despite the autofluorescence from the biological tissues. Classical least squares is an algorithm that provides signal isolation with good reproducibility [312]. It has been demonstrated to improve SERS detection and imaging with emphasis on multiplexed assays (Fig. 17a and b) [307, 311]. Recently, Oliveira et al*.* [313] showed that by using CLS method to resolve the mixed spectra from each pixel of the Raman map, SERS tags were able to differentiate a positive from negative response with high specificity and accuracy (Fig. 17b). To train a multiplex assay to detect a particular condition, Sánchez-Purrà et al*.* used non-negative least squares (NNLS) coupled with linear discriminant analysis (LDA) to discriminate between SERS tags and train the system to be able to associate different tags ratios [294]. This approach surpasses the conventional peak integral where even small overlaps can be falsely recognised as SERS signal.

SERS quantification irreproducibility at ultralow concentrations (< 1 nM) can also be improved by MCR methods. To quantify SM-SERS signals, a digitalisation procedure can be implemented by non-negative matrix factorization with alternating least squares algorithm (NMF-ALS) that embraces the stochastic characteristics of the SM-SERS intensity fluctuations and generates a calibration curve by counting the SM events [314].

The mentioned methods, exploit machine learning (ML) in that the chosen algorithm is acquiring knowledge by extracting features from raw data. Then, the obtained knowledge is used to make informed decisions when dealing with a problem. Among ML methods, convolutional neural network (CNN) is a popular method for spectral analysis due to its prediction accuracy with medium or large data sets that outperforms standard classification algorithms used in chemometrics [315]. SERS-CNN method has been proved to be suitable to identify and discriminate small DNA damages [316], tumour and normal cell’s metabolites [317], neurotransmiters [280] and even able to identify microbes at a single-cell level [271], all with 98% or higher levels of accuracy overcoming signal interferences and without optimisation of test conditions. Using an occlusion-based Raman spectra feature extraction, Lu et al*.* expand the CNN model applicability to visualise the essential features of Raman spectra that CNN model used for discriminating different species [271]. More than label-free SERS applications, CNN proved suitable to address the challenge of quantifying sub-nanomolar analyte concentrations [318]. Furthermore, the model was generalisable to different analytes by transfer learning implementation [318]. A scheme of the experimental and analytical steps used to ‘train’ the data mining method to build a predictive model is represented in Fig. 17c. These works open possibilities towards multiplexing SERS-tags application even in a single-molecule detection system.

Deep learning (DL) applied into SERS field has also shown promise specially in healthcare applications [319]. Without the need for specific pre-processing or feature engineering, DL methods can automatically extract and transform the features into a higher-level representation. Notably, Shin et al*.* demonstrated that a residual neural network (Resnet)-based DL model was possible to be trained with SERS data set of exosomes derived from normal and lung cancer cell lines and then used to predict the cancer stage in patient plasma samples with an accuracy of 95% (Fig. 17d) [319]. In another case, deep neural network was used to recognise specific deoxyribonucleic acid targets reaching 95% accuracy [320].

However, DL and ML-based SERS approaches depend on overcome the irreproducibility in SERS data. Large variance in the data set increases the variance in predictions, which may have negative repercussions in semiquantitative or quantitative analysis. These problems may be mitigated with implementation of algorithms such as multi-objective evolutionary algorithm (MOEA) [321]. Also, the application of these methods require validation with traditional approaches to be accepted into practical use. This poses a problem considering that deep learning algorithms that tackle biosensing problems may require thousands of examples which represents an enormous barrier for the development of SERS in a clinical setting.

Nevertheless, ML methods have an ability to interrogate appropriate nonlinear dependencies for complex data set offering the inimitable possibility of solving pressing challenges in the area of SERS bioanalysis and making it a promising methodology for use in clinical samples.

### Automation through microfluidics

Automation is a requirement to bring the SERS immunoassay for point-of-care sites. Microfluidics can help to accomplish that need through the integration of several protocols into one single device. In terms of disease diagnosis, integrating the entire process, from blood collection to the detection, is highly desirable. Non-invasive specimens such as saliva and urine have been proposed as an alternative to blood samples in the case of malaria, but have yet to be validated [323]. Hence, the device would profit from providing a way to simplify the pre-treatment need to extract the analyte for end users.

**Fig. 18:**
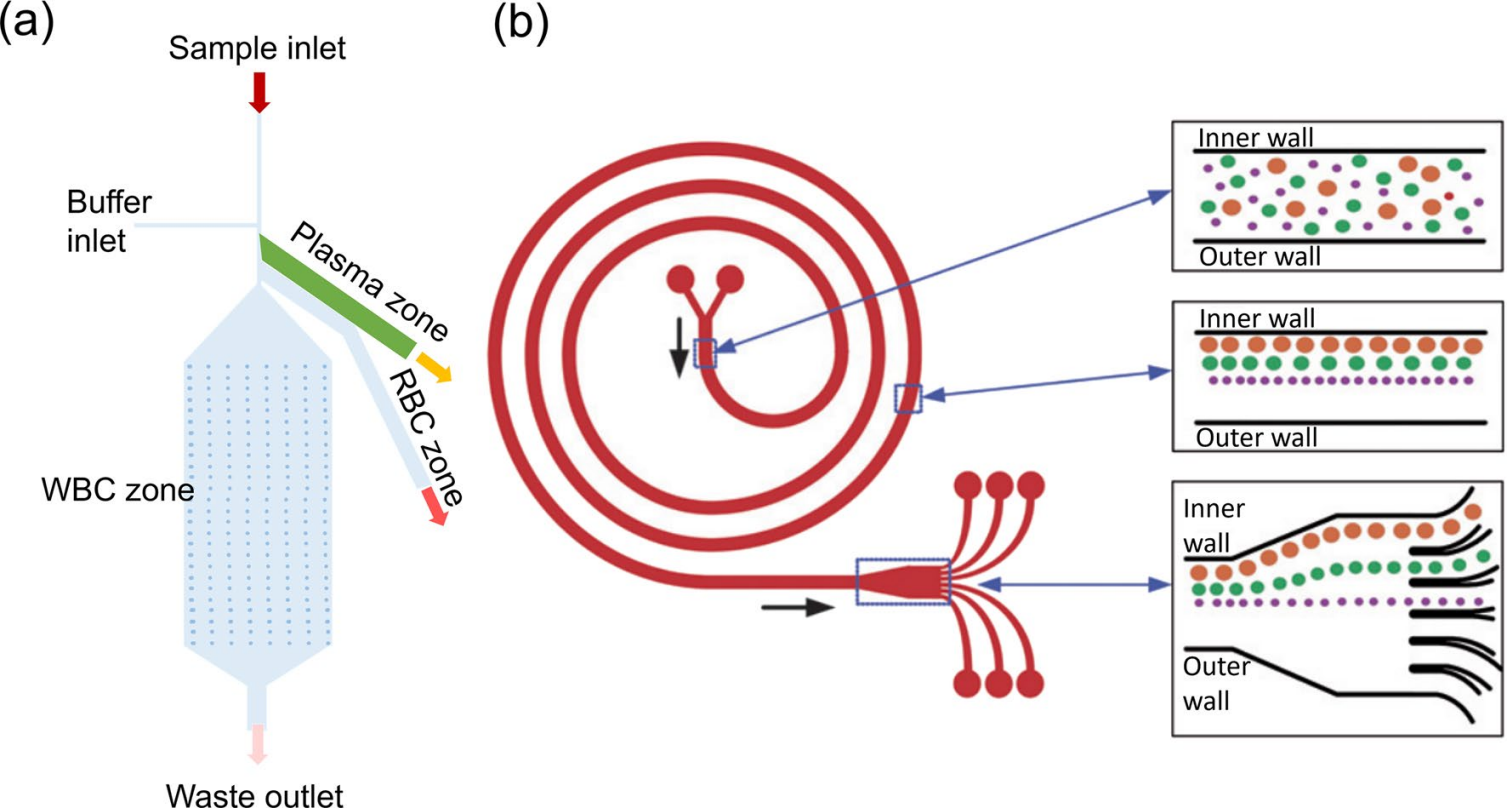
Passive separation method for automated microfluidic system. **a** Scheme of the microfluidic device for blood separation. The blood sample flows to the bifurcation region and plasma and RBC were extracted to plasma and RBC zone, respectively. WBC flows to the main channel and are trapped in the WBC zone. Adapted from [324]. **b** Scheme of the spiral microparticle separator. Randomly dispersed particles of different dimensions are introduced in the inlet and along the spiral microchannels will dispersed at different equilibrium positions along the inner wall due to a combination of inertial lift and Dean drag forces. Then the individual particle streams are extracted by a multiple outlet design. Adapted from [325]

Passive separation methods represent a valuable option, since they do not require any cell labelling or sophisticated microfabrication process [324]. Hence, these systems are more cost-effective and adequate for separating blood cells from plasma. Examples of possible apparatus are illustrated in Fig. 18. The separation mechanism can be based on the bifurcation law, or the Dean coupled inertial migration effect. The first states that, within a laminar flow profile, the cells at a point of bifurcation will have a propensity to flow into the microchannel with a higher flow rate (Fig. 18a) [324]. Consequently, the adjacent channel with a lower flow rate will contain a purified, cell-free fraction of plasma. The latter (Fig. 18b) is achieved with particles flowing in a spiral microchannel with a rectangular cross-section [325].

Another relevant development that could bring the biosensor closer to the POCT requirements, is the synthesis of SERS tags inside the microfluidic device, which would rely on the efficient storage of the reagents used for their design. This could also contribute to a more reproducible and standardised conjugation. A few examples of micro-reactors exist in the literature [20, 248], but significant efforts are still needed to develop an integrated high-throughput platform for synthesis of nanostructures, material evaluation, and biofunction screening. Only when these procedures are completely unified, can a full POCT biosensor be realised.

Furthermore, once the adequate automation coupled with miniaturisation and reliability of microfluidic devices is achieved, handheld Raman spectrometers can be used for SERS measurements. The emergence of portable Raman spectrometers with a wide range of laser wavelengths and sampling conditions, as well as online data-processing methods has made them suitable for POCT, thus making SERS-based microfluidic devices a reality in a close future [21].

### Lab-on-paper SERS assays

Among the several SERS microfluidic assays, paper-based methods are very promising for POCT sites as they provide a rapid, low cost, portable and sensitive method.

Nitrocellulose and cellulose acetate, are the paper-based substrates commonly used in lateral (LFA) and vertical (VFA) flow assays [326]. LFA and VFA have several advantages, such as being all-in-one devices, rapid, low-cost, and easy to assemble and use [326]. These biosensors can be used in many areas, such as healthcare, agriculture, food contamination, forensic science, and animal health [327].

**Fig. 19:**
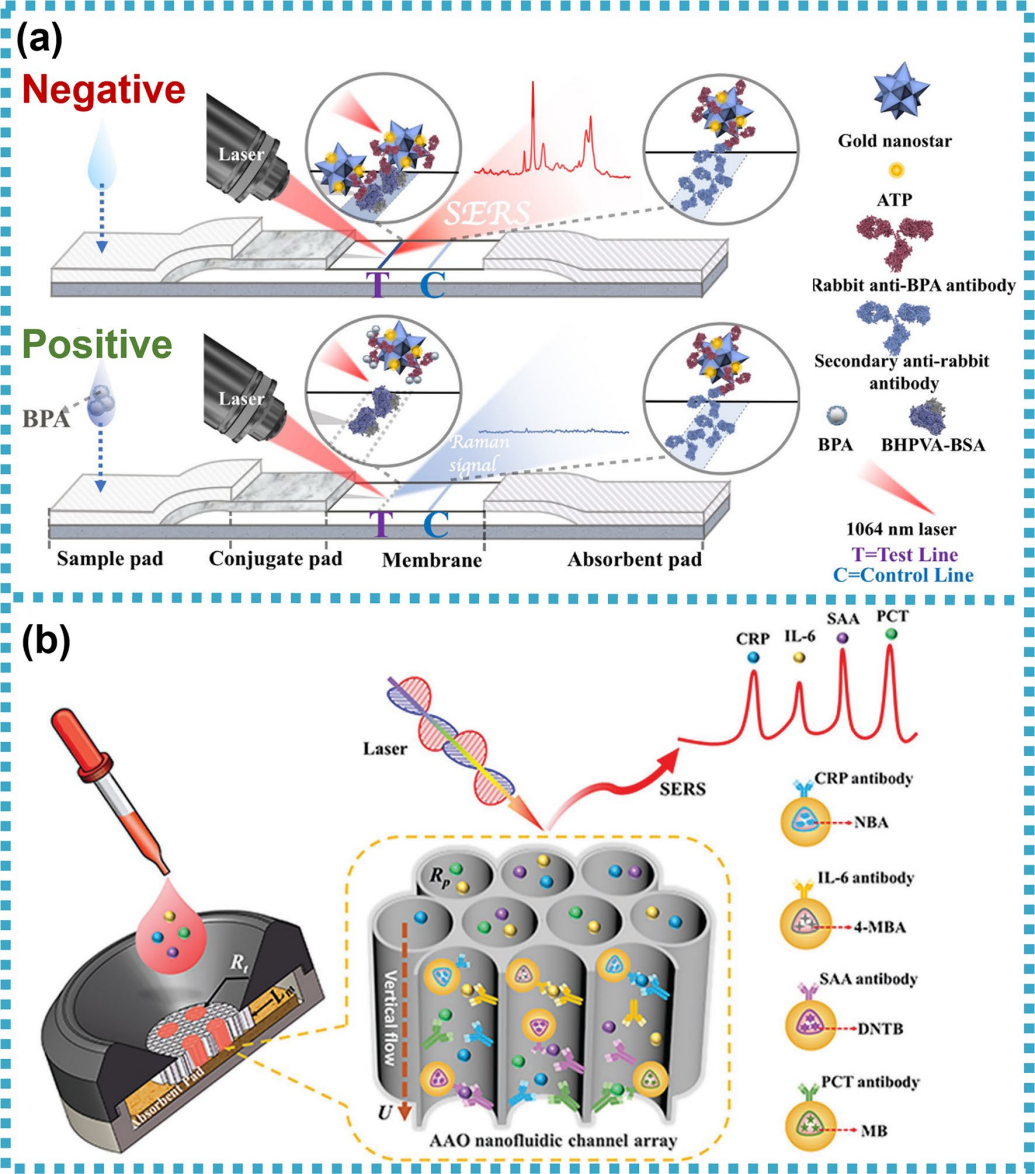
**a** BPA SERS-LFA test kit using a competitive format. Adapted from [338]. **b** Nanoporous Anodic aluminium oxide -based multiplex VFA for the detection of four inflammatory biomarkers with Raman dyes encoded core–shell SERS tags. Adapted from [331]

Flow assays usually have two main sensing strategies, competitive or non-competitive (sandwich) assays. The first one is normally used for small target molecules (< 1 kDa), which cannot bind to two antibodies simultaneously [326, 328]. In competitive assays, the small analyte competes with the tag for the receptor in the test line and thus, the intensity of the signal is negatively correlated with concentration of the target antigen (Fig. 19a). The sandwich assay is more suited for mid- and big-size analytes (> 1 kDa) (*e.g.*, proteins and antibodies), where the target molecule is captured by the tag in the conjugate pad, and the target tag complex is captured by the target-specific ligand on the test line. Thus, for the sandwich assay, the signal intensity is positively correlated with the analyte concentration [326, 328].

In these LFA biosensors, the target analyte in the sample is specifically captured by tags in the conjugate pad, that consists of nanoparticles (*e.g.*, AuNPs) functionalised with bioreceptors that recognise and capture the target (*e.g.*, antibodies or aptamers). The sample continues moving towards the adsorption pad, and the complex formed (*e.g.*, AuNPs-antibody conjugated with the target) can be captured by the target-specific ligand that is immobilised on the test line. The control line has a specific anti-immunoglobulin antibody to capture tags independently if the target is present or not and is used to test whether the assay is functioning properly [329].

VFA represent an alternative to LFA, with similar components but with a higher multiplexing capacity [330]. The main difference is the liquid flow direction, which runs vertically on the membrane in VFA. Unlike the capillary forces of LFA, VFA may depend on external forces, such as pumps whereby the pore size of the membrane, and the flow rates are modified to improve the sensitivity of the assay [330, 331]. Usually, the VFA is constructed as a non-competitive assay with two different designs [332]. One design is assembled with sample pad, conjugate pad, membrane, and absorbent pad, from bottom to top, and the sample is placed on top (Fig. 19b) [331]. In the other design, all the reagents are placed vertically one by one in a single membrane [327].

Several SERS-based LFAs and VFAs have been developed to improve the sensitivity and the multiplexing abilities of the traditional colorimetric flow assays. In fact, it has been shown that the sensitivity of these test strips can be improved by 2–3 orders of magnitude and precision when SERS is used [329]. As an example, Lin et al*.* [333], developed a competitive SERS-based LFA with AuNSs functionalised with 4-aminothiolphenol and an antibody to detect bisphenol A and 4,4-bis(4-hydroxyphenyl) valeric acid-bovine serum albumin. As a result, the Raman intensity was negatively correlated with the concentration of bisphenol A, achieving a limit of detection of 15 ppb by colorimetric detection and 0.073 ppb by SERS analysis. Moreover, the SERS-based flow assays can be designed for single or multi-targets detection, whereby the latter can be accomplished by several line tests or multiple Raman-labelled SERS probes for different targets [329]. For instance, Sánchez-Purrà, et al*.* [334] developed a SERS-based LFA to detect Zika and Dengue biomarkers, by visual and through Raman spectroscopy. This assay was performed with AuNSs with two Raman reporters, MBA and 1,2-bis(4-pyridyl)ethylene. The multiplex ability was improved upon by Zhang et al*.* [335] who developed a competitive assay, with which it is possible to detect six major mycotoxins simultaneously, aflatoxin B1, zearalenone, fumonisin B1, deoxynivalenol, ochratoxin A, and T-2 toxin.

To demonstrate the higher multiplexing ability of VFA, Chinnasamy et al. [336] developed a VFA with 1,480 binders, high sensitivity, and less than 10 min of assay. In another example of VFA, Chen et al*.* [337] developed a SERS-VFA for multiplex prostate cancer biomarker detection, whereby, on the same membrane, it was possible to detect different target molecules separately. Notably, however, although VFA brings advantages in multiplexing, the operation is complicated, which limits the potential of this test [329].

The LFA market has seen continuous growth, especially during the SARS-CoV-2 pandemic due to the need of rapid diagnostic tests [334].Nevertheless, SERS-based paper biosensors still require improvements in SERS labels synthesis that ensure the tests reproducibility, the possibility of scaling-up the SERS-based assays, and the sensitivity needs to be demonstrated by portable Raman spectrometer to show its real-world applicability [329].

## Conclusions

The design of nanosystems is already revolutionising the biosensors field and SERS has been established as a powerful and versatile technique that offers higher sensitivity, signal specificity, and unparalleled multiplexing capabilities over traditional detection and imaging models. Thus, it has been exploited for applications from ultrasensitive and multiplex detection systems, to targeted controlled drug delivery and in vivo imaging systems.

Despite the increase in several applications in environmental, food safety, and medical healthcare areas, SERS has still not reached its full potential. Its widespread use in detection systems and their commercial attractiveness has been hindered due to batch-to-batch irreproducibility, the need for calibration and lack of large-scale synthetic procedures. Moreover, selecting the most suited SERS tag production strategy is not trivial and due to the wide variety of inorganic NPs and biomolecules available, no universal methodology exists. The striking features that make the nanomaterials so appealing such as the tunability they offer in terms of size, shape, charge, surface area, colloidal stability, density, and others, makes difficult to transfer one protocol that works for a particular NP to another different system. Similarly, RRs and biomolecules vary in terms of size, chemical composition and 3D complexity, among others, which increases the different possible combinations of functionalisation and bioconjugation protocols. Thus, each case needs optimisation, and a considerable amount of research has been dedicated to developing innovative SERS tags considering the intended application. Significant efforts have been devoted to fabricate nanostructures with high level of uniformity and reproducibility which can help to tackle the frequent problem of Raman intensity variations. Interestingly, this progress is somehow mitigated by the comparatively underdeveloped RRs field. Thus, although the functionality of these SERS tags is unquestionable, they are probably capable of far more. Moreover, the heterogeneity found within the NP materials and the resulting bioconjugates concerning orientation and attachment ratio remains a crucial concern. Hence, the need for non-heterogeneous and site-specific chemistries within the bioconjugation techniques is critical and the development of commercially available SERS tags together with standardised conjugation protocols will certainly play a role in the future.

Furthermore, performance comparison and cross reference the spectral analysis of SERS with existing well-established techniques already on the market is also important. While data reproducibility is crucial in research laboratories, clinical applications should conform to more rigorous standards. Therefore, studies must be extended to clinically relevant scales for meaningful statistical analysis. To address this hurdle, machine learning approaches stands as a very promising tool. The advent of machine learning, beyond implementation of statistical data analysis, will surpass the quantification and identification of multiple analytes within complex mixtures, as to evolve into approaches able to make predictions or decisions without being explicitly programmed to do so.

Associated to nanosystems for biosensing tools, comes microfluidic devices, to provide outstanding miniaturised analytical platforms. The world's growing and ageing population is causing a burden in the healthcare system. Consequently, the healthcare industry aims to replace the diagnostic tools that require a sophisticated infrastructure into portable alternatives, practicable at healthcare centres and in-home care, *i.e.,* POCT sites. These alternatives will help to bring effective and quality primary care on a global scale.

The quest for accurate POCT diagnostics with microfluidic-based technologies has been somehow hindered by the use of PDMS, due to its ultra-high precision machining requirements that cannot translate these research prototypes to mass production in a cost-efficient manner. Therefore, microfluidics is mainly confined to the laboratory stage of scientific research with low maturity products in the market. Chemically compatible and optically transparent materials that allow simple and rapid fabrication while eliminating the need for PDMS will certainly help in the achievement of microfluidics in POCT diagnostics. As stated before, PS present several attractive features for the fabrication of a microfluidic device as demonstrated by this work. Nevertheless, the fabrication of multiple protocols integrated into a single microfluidic chip may be dependent on a thermoplastic with built-in actuators such as microvalves and micropumps which can pose some challenges during implementation, as their inherent high rigidity and Young's modulus makes the fabrication of thin elastomeric membranes challenging. Recently, further efforts have been made with actuators based on styrene-ethylene/butylene-styrene, Teflon, PMMA combined with TPU or even using only PS, without complicated fabrication processes and increased cost [242].

Several examples of integration of biomolecular processes in microfluidic devices have been reported in literature [21]. Still, the integration of reagents in a consistent, low-cost, and precisely controlled device, while avoiding cross contamination, maintaining ease of operation, and with an appropriate shelf-life for commercial ends, is so far absent. Furthermore, whilst integration is fundamental for a portable device, the range of operating conditions has been largely overlooked by researchers. Temperature and humidity change considerably among the potential POCT sites and data on thermal stability of most developed biosensors has yet to be reported. When these hurdles are finally conquered, microfluidics devices will represent a sensitive, portable, and independent diagnostic tool and then, their commercialisation to an economy of scale can begin. Paper-based microfluidics can be relevant as unexpensive yet sensitive, alternative but still needs some efforts in using paper-biofunctional material hybrids.

As a final thought, a thorough Life Cycle Analysis should be undertaken to assess the possible impacts on the environment and human health from an exposure of the entire life cycle of a device. The development of novel nature-based materials that come from sustainable and economically efficient processes represents an ever more preeminent challenge, as highlighted by the European Green Deal and the United Nation's Sustainable Development [339]. While the concepts of simplicity, sustainability, versatility, and low-power consumption were always present during this work, as the society evolves towards a more environmental conscious mindset, exciting sustainable technological advances are made and will unquestionably define our future.

In conclusion, SERS field is growing towards becoming an important tool for detection and personalised diagnosis. Only when SERS tags standardisation, meaningful statistical analysis, and cost-efficient large scale microfluidic device fabrication coupled with portable Raman spectrometers are achieved, can SERS-based microfluidic devices for POCT sites become a reality in biosensors market. This almost fifty-years old technique still has some obstacles to conquer and might become a standard tool working together with others bringing novel and exciting features.

## Data Availability

Not applicable.
